# Dimer Interface of the Human Serotonin Transporter and Effect of the Membrane Composition

**DOI:** 10.1038/s41598-018-22912-7

**Published:** 2018-03-23

**Authors:** Xavier Periole, Talia Zeppelin, Birgit Schiøtt

**Affiliations:** Interdisciplinary Nanoscience Center, Department of Chemistry, Aarhus University, Aarhus, Denmark

## Abstract

The oligomeric state of membrane proteins has recently emerged in many cases as having an effect on their function. However, the intrinsic dynamics of their spatial organization in cells and model systems makes it challenging to characterize. Here we use molecular dynamics (MD) simulations at multiple resolutions to determine the dimer conformation of the human serotonin transporter (hSERT). From self-assembly simulations we predict dimer candidates and subsequently quantify their relative strength. We use umbrella sampling (US) replica exchange MD simulations for which we present extensive analysis of their efficiency and improved sampling compared to regular US MD simulations. The data shows that the most stable hSERT dimer interface is symmetrical and involves transmembrane helix 12 (TM12), similar to the crystal structure of the bacterial homologue LeuT, but with a slightly different orientation. We also describe the supramolecular organization of hSERT from a 250 μs self-assembly simulation. Finally, the effects of the presence of phosphatidylinositol bisphosphate or cholesterol in the membrane model has been quantified for the TM12-TM12 predicted interface. Collectively, the presented data bring new insight to the area of protein and lipid interplay in biological membranes.

## Introduction

Oligomerization of integral proteins in crowded cell membranes has recently emerged as a dynamic process that could be used in cells as a regulatory mechanism. Such mechanism could act through the cell’s ability to translocate (traffic) proteins between organelles and compartments of the cell membrane^1^, thereby changing the environment of the protein, which, in turn, would affect its oligomerization propensity and thereby affect their function^2^. Alternatively, a direct modification of the confined lipidic environment of the protein could be used to affect protein oligomerization and function.

Neurons and the space through which they communicate, the synapse, are the stage of a multitude of such processes, contributing to the synaptic plasticity^2^; eg. GABA^2^, NMDA^3^ and AMPA receptor trafficking^4,5^, DAT function and trafficking^6^, SERT partitioning and function^7^, trafficking and oligomeric state^8^, and generally neurotransmitter:sodium transporters^9,10^. The functional role of protein oligomerization in the synapses is however still unclear in most cases.

In this study we focus on monoamine transporters (MATs). MATs are proteins located in the pre-synaptic plasma membrane and assure the re-uptake of neurotransmitters serotonin (5-hydroxytryptamine, 5HT), dopamine (DA) and norepinephrine (NE) from the synaptic cleft into the pre-synaptic neuron. Neurotransmitter re-uptake is a key step for proper neurotransmission and MATs dysfunction has been associated with multiple neuronal disorders such as depression^11^, ADHD^12^, Parkinson^12^ and addiction^13^, making MATs the primary targets for treatment of mental disorders^14^.

Notably, MATs have been shown experimentally to form oligomers in the synaptic plasma membrane^15^, but it is unclear which regulatory or functional role these may have. Many lines of evidence suggest that DA and 5-HT transporters (DAT and SERT, respectively) can form a homotetramer presumably consisting of two dimers^16–19^, but recent experiments have also shown that SERT exists in many different oligomeric states including monomers^8,15^. As for today we know that DAT oligomer formation is a prerequisite for ER export and integration into the plasma membrane^20^. DAT also has a cooperative effect on inhibitor binding and substrate transport in the individual protomers^21^. Supporting this idea, recent simulations indicate that DAT collective motions resemble more the conformational changes associated with transport when it is analyzed as a dimer than as a monomer, suggesting that DAT would be a more efficient transporter in a dimer conformation^22^. Access to the structures of MAT dimers and higher order oligomers at the atomistic resolution appear therefore timely to guide and inspire the design of new experiments.

**Figure 1:**
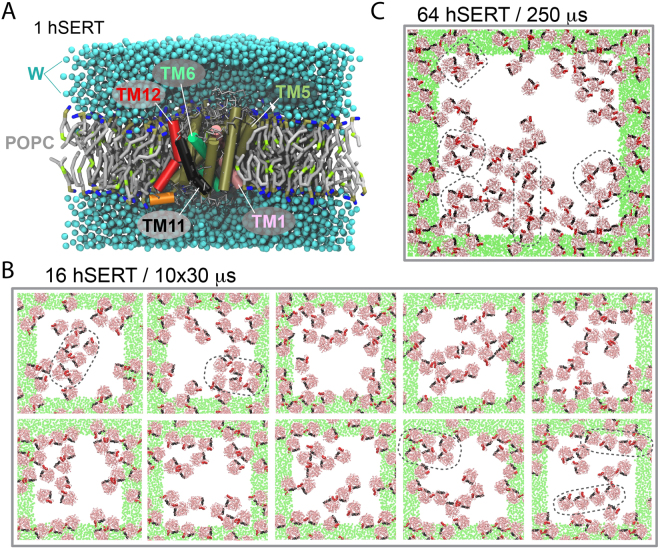
Systems simulated. hSERT was embedded into a series of lipid bilayers (POPC, grey tails, and tan and blue headgroups) and solvated in water (W, blue). (**A**) One copy of hSERT embedded in 380 POPC molecules; some transmembrane (TM) helices are highlighted for convenience, and lipid and water molecules are not shown to feature the transporter. (**B**) 16 copies of hSERT inserted in a POPC lipid membrane. The final configuration of ten self-assembly simulations of 30 μs each are depicted. (**C**) Configuration of 64 copies of hSERT after a 250-μs self-assembly simulation. In (**B** and **C**), the systems are viewed from the top of the membrane, a 1:99 protein:lipid ratio was used, TM11 (black) and TM12 (red) are highlighted, the solvent and the lipid molecules are omitted for clarity, hSERT’s backbone trace is shown in pink, the green areas represent the periodic images of the central cell (white). In (**B** and **C**), a few illustrative aggregates are highlighted in black.

A few studies have provided important clues to MATs dimer conformation but yet no consensus has emerged. Cysteine crosslinking experiments suggest that transmembrane helix (TM) 4 (Cys243) and TM6 (Cys306) are involved in the formation of a symmetrical dimer in DAT^16,23^. The overall organization of MAT TMs is given in Fig. 1A using hSERT-numbering. A DAT dimer interface involving TM6 was also more recently suggested by a combination of crosslinking, mutagenesis and computational experiments^24^. In addition, four tandem repeats of a leucine heptad (LX_6_LX_6_LX_6_L), a structural motif which underlies oligomerization in phospholamban^25^, is present but incomplete in MATs’ TM2. A mutational study of this repeat in DAT suggested that TM2 should be involved in a symmetrical dimer interface^26^. However, in the crystal structures of hSERT^27^ and dDAT^28^, this repeat is buried within the core of the proteins preventing it from participating directly in an interface^29^. Furthermore, in the case of SERT, FRET experiments have shown that the fragment pairs TM1/TM2 and TM11/TM12 might represent symmetrical intermolecular interaction sites^30^. A GXXXG motif in SERT’s TM12, but not conserved across MATs, was proposed to participate in the interaction site in the TM11/TM12 pair^9^. But again, visual inspection of the recent hSERT crystal structure revealed that this repeat is not exposed to the membrane. Nevertheless, an interface involving TM11/TM12 would be in agreement with the crystal structure of the MAT’s bacterial homologue, LeuT, which when resolved as a symmetrical dimer involves TM9, TM12 and extra-cellular loop 2 (ECL2) at the interface^31^. LeuT has also been resolved as a monomer. Overall, current data do not support a unique dimer model for all MATs, but instead suggests that the different transporters may have different interfaces.

Furthermore, mounting evidence has stressed the importance of the lipid composition in biological membranes for the formation and stabilization of protein oligomers^32–36^, a factor that could be used to regulate oligomerization in cells and thereby the function of proteins. In the case of MATs, the oligomeric state of hSERT has been shown to evolve from a dynamic exchange of the transporters between the multiple oligomer types in the ER to a more static distribution in the cell membrane^8^. Although the presence of phosphatidylinositol bisphosphate (PIP2) in the cell membrane is key to this transition, hSERT assembly also occurs in the absence of PIP2^8^. The role of PIP2 remains to be determined but it was suggested to interact with the N-terminus of hDAT^37^. More recently, DAT nanodomains where found sensitive to cholesterol (CHOL) depletion and potentially implicated in modulating dopaminergic neurotransmission^6^. Also recently, mass spectroscopy studies underline the importance of lipids in the stabilization of LeuT^36^ dimers. In this study, cardiolipin stabilizes the LeuT dimer in addition to six phospholipids most likely bound at the interface^36^. Alternatively, lipids can affect protein’s function through a direct interaction as we have recently shown recently experimentally for hSERT^38^ and computationally for hDAT^39^.

Here we determine the structure of the hSERT dimer and characterize its supramolecular organization in a membrane environment. We apply our approach developed previously to determine the relative strength of membrane protein interfaces^40^, which consists in the use of coarse grain molecular dynamics and relies on the determination of potentials of mean force (PMF) of dimer candidates extracted from self-assembly simulations. The method is herein improved by replacing regular umbrella sampling (US) simulations by US replica exchange molecular dynamics (REMD) simulations^41^, which we show considerably improved convergence for. The dimer structure of hSERT extracted with this enhanced method shows an interface centered on TM12 but with a different orientation of the protein core than seen for LeuT^31^. We also estimated the effect of PIP2 and CHOL on the strength of this dimer interface and infer on the mechanism by which these lipids might affect the interface.

## Results

### Four main interfaces are formed during the self-assembly simulations

Ten self-assembly simulations of 16 hSERT molecules and one with 64 molecules embedded in a pure POPC lipid membrane collectively show that hSERT forms dimers and higher order oligomers with multiple interfaces on a μs time scale (Fig. 1B,C). Each hSERT monomer is on average in contact with two other monomers (Figs 1B,C and S1) resulting in a supramolecular organization with different sizes of aggregates. Some align almost linearly, others form small aggregates and a few combine both to form small stretches of rows-of-dimers (Example are encircled in Fig. 1B,C).

**Figure 2:**
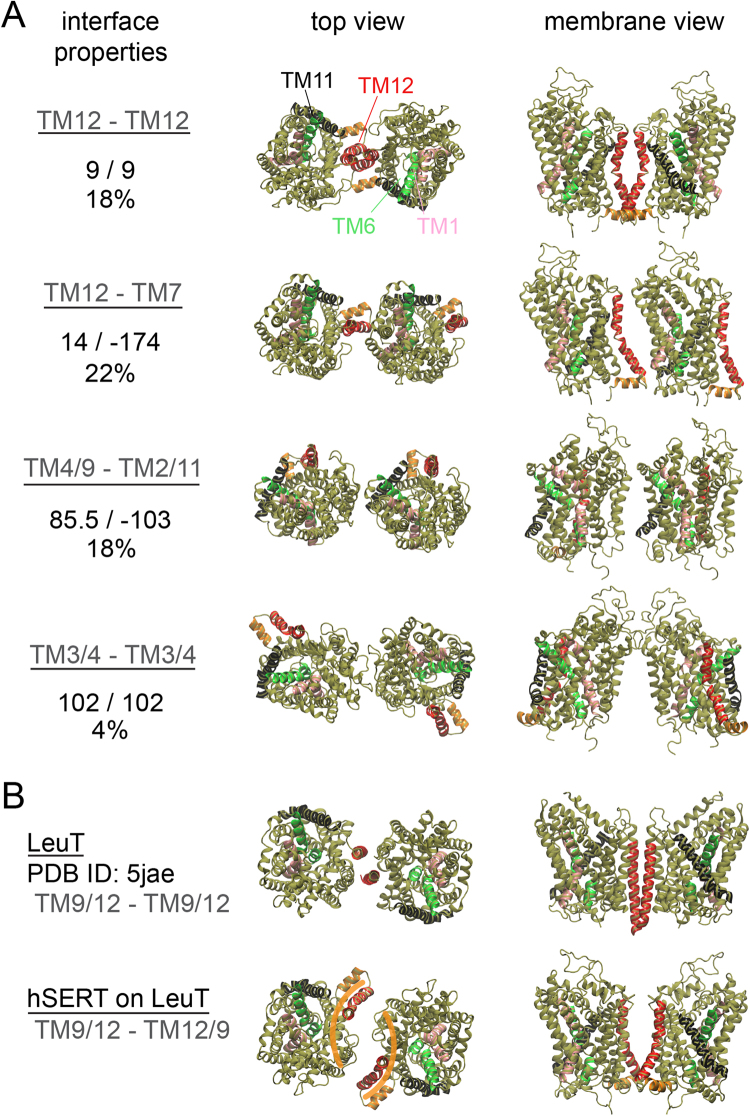
hSERT interfaces. (**A**) Representative structures of the four most populated interfaces formed in the self-assembly CG simulations of hSERT in a POPC membrane (See text for details). From top to bottom: TM12-TM12, TM12-TM7, TM4/9-TM2/11 and TM3/4-TM3/4. For each interface the values of the ϕ1 and ϕ3 angles used to define their relative orientation in the VBA (See Figure S26 for details) and their contributions to the first 10 clusters are indicated. Atomistic structures of hSERT were fitted onto the CG configurations. (**B**) Top: Interface of LeuT (bacterial homologue of hSERT) as found in a crystal structure. Bottom: hSERT fitted on LeuT. TM12 (red) was omitted during the fitting procedure. One can appreciate the resulting different locations of TM12. The orange bananas illustrate the complemented crescent moons of the interface. The color code is as in Fig. 1A.

A cluster analysis of hSERT dimers formed during the ten repeats of self-assembly simulations identified four main hSERT dimer interfaces (Fig. 2A). These conformations were found within the first 10 clusters and had more than 3% of the clusters analyzed (Figure S2). The two predominant clusters involve TM12 and account for 40% of the interfaces analyzed. In the first cluster hSERT forms a symmetric interface (TM12-TM12) and in the second cluster an asymmetric one is formed, where TM12 engages in a contact with TM7 in the second monomer. The presence of TM12 at the hSERT dimer interface is in line with experimental studies pointing to its contribution to the dimer interface^30,31,43^. The third cluster is an asymmetric dimer, which involves TM4/9 and TM2/11. The forth cluster is symmetric and the interface is primarily formed in the aqueous phase, involving the extracellular ends of TM3 and TM4 and the connecting loop, ECL2 (Fig. 2A).

This organization may be compared to the case of other proteins. Rhodopsin, a prototypical G protein-coupled receptor (GPCR), also forms on average two contacts per monomer but linear arrays predominate their supramolecular organization in self-assembly simulations^40^. Similar differences of protein organization in lipid membranes were reported for model proteins in response of the protein class and shape, hydrophobic mismatch and membrane curvature^42^.

Notable is the similarity of the symmetric interface using TM12 (cluster 1) with the interface formed in crystals by the bacterial homologue of SERT, LeuT^31^. In both cases TM12 is the main contribution to the interface (Fig. 2B). However, the two interfaces differ. First, the contacts between TM12 change. While in hSERT the two TM12 seem to wrap around each other taking advantage of a kink in the middle of the helix, in LeuT TM12 is straight and the interface involves another helix, TM9, in contact with TM12 (Fig. 2B). Second, the position of TM12 relative to the rest of the helical bundle of the transporter (TM1–11) differs in LeuT and in hSERT. This difference leads to a distinct orientation of the proteins helical bundle relative to the two TM12 interfaces. This difference is apparent when the hSERT helical bundle (TM1–11) is superimposed on LeuT (Fig. 2B). This operation is equivalent to a rotation of hSERT around the membrane normal, opening a large space between the monomers, which then lack direct contacts. This operation also rotates TM12 away from the interface then mainly facing TM9 (Fig. 2B). The relaxation of this interface is described later in this manuscript.

### PMF: efficiency and convergence of US-MD and US-REMD

To determine the relative strength of hSERT interfaces found in the self-assembly simulations we have calculated the potential of mean force (PMF) using the umbrella sampling (US) method^44^ coupled with replica exchange molecular dynamics (REMD) simulations, also called window or umbrella exchange MD simulations^41^. In this section we present an analysis on the efficiency and convergence of such simulations and we compare them to conventional US-MD simulations.

In the determination of a PMF using the US technique, the starting conformation of the system used at each umbrella is key because the conformational sampling might be affected in cases where the free energy surface is complex and contains multiple wells. Convergence is difficult to attain even in simple cases^40,45–48^. In principle, by allowing the system to walk the reaction coordinate (umbrella) space US-REMD should allow for a better sampling and therefore a faster convergence than US-MD^41^.

**Figure 3:**
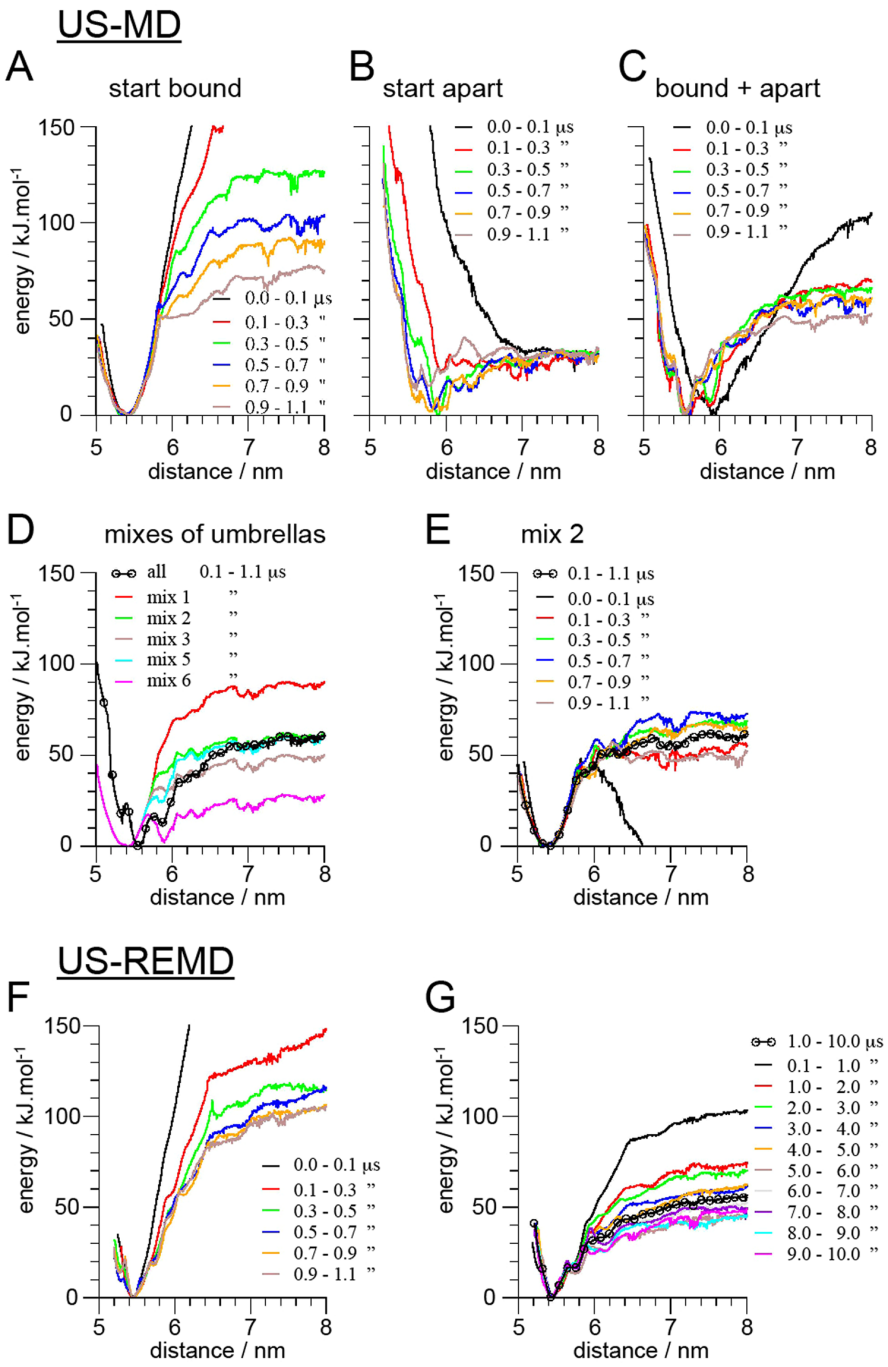
Convergence of umbrella sampling (US) MD simulations. The convergence is illustrated by following the evolution of the potentials of mean force (PMF) for one of the most populated interfaces: TM12-TM12. PMFs were obtained with (**A**–**E**) a conventional US-MD approach for membrane proteins or (**F**,**G**) with an US-replica exchange molecular dynamics (REMD). The different curves in each panel represent the PMF obtained using different time window as indicted in the color legends. The US-MD were either started from a bound (**A**) or unbound state of the proteins. In (**C**) both (**A**) and (**B**) were combined using all the umbrella windows, while in (**D**) subsets of umbrellas were used (see Figure S4 for details of the windows used). In (**E**) the convergence of the mix 2 is shown. In (**F** and **G**) the proteins were initially bound.

We compared US-MD simulations starting with the proteins bound or unbound in all umbrella windows. Their progression over a 1.1 μs simulation shows that both have difficulties to converge (Fig. 3A,B). While the simulation started from bound proteins has difficulties to sample the umbrella windows where the proteins are at the boundary between bound and unbound states (shoulder forming at ~5.8/6.0 nm as the simulation time increases in Fig. 3A), the simulation started from unbound proteins fails to sample the bound state (minimum of the PMF at 5.4 nm). These limitations are respectively due to the energy barrier needed to separate the proteins leading to a sudden separation of the proteins, and the trapping of lipids at the protein interface preventing them from exploring the minimum. Both simulations lead to major errors in the PMF as the system may adopt very unrealistic conformations and remain trapped for extended periods of time. Interestingly, the blind combination of both simulations leads to a reasonable PMF (Fig. 3C). On a technical note, it is actually difficult to understand how WHAM is able to converge since for many umbrella windows the histograms are significantly different in the two simulations (Figure S3).

Similar issues were observed in our previous study of rhodopsin in which case up to 20 μs per umbrella simulation were not sufficient to overcome the energy barriers trapping the system^40^. We therefore used a strategy consisting of mixing umbrella simulations started from bound and unbound conformations. While, as discussed above, one can blindly combine all the umbrellas (see Fig. 3C for the example of hSERT). However, this approach typically requires the determination of an appropriate mixture of bound and unbound umbrella simulations to calculate an accurate PMF. PMFs are indeed extremely sensitive to the set of windows given to WHAM (Figs 3D,E, S3). Although, when carried out with extreme care this approach can provide reliable results^40^, it is a tedious task and will always be sensitive to the user’s understanding of the system and therefore undoubtedly subject to bias. We illustrate the practice of that sensitive approach on one interface of hSERT (Figs 3D,E, S4).

US-REMD simulations seem to offer an ideal practical solution to this issue. The systems or replicas are allowed to walk through the umbrella space (protein interfacial separation) and thereby explore different conformations. The proteins should bind and unbind and the lipids should get trapped and un-trapped as the distance between the proteins varies. We first tested the efficiency of US-REMD simulation as a function of the frequency of exchange trials between umbrella windows. Values of 20, 200 and 2000 ps were compared by monitoring the evolution of the PMF calculated using 0.1 μs time windows over a period of 0.9 μs (Figure S5). Although theses simulations are not converged themselves, one can appreciate that a 20 ps interval between exchange trials is the most efficient. It allows the system to overcome barriers faster. In the cases of 200 and 2000 ps between attempts of exchange of replicas the PMF seems to get trapped for longer periods of time than when attempts are tried every 20 ps (Figure S5). An interval of 20 ps between exchange trials is used in all the following simulations.

We then tested whether starting from a bound or an unbound conformation of the protein would also impact US-REMD as described above for US-MD and reported previously for US-REMD applied to more simple systems^48^. The comparison of two US-REMD simulations in which all replicas are started from either a bound or an unbound state shows that it does matter in a similar manner. Over a 2.6 μs period, the US-REMD simulation started from an unbound configuration fails to sample the minimum or the bound state (Figure S6), due to lipid molecules being trapped at the interface. This limitation leads to a very rough and apparently weak binding similar to the US-MD results. In contrast, the US-REMD simulation started with bound proteins produces an apparently more realistic profile (Figure S6). The distances at the minimum are well sampled but also the distances corresponding to protein separation, removing the shoulder observed in the US-MD PMF (Fig. 3A). It is unfortunately not currently possible to extend these two simulations so that both curves would converge, similar to what Domanski *et al*. were able to do on a more simple system^48^. We expect the computational cost to be prohibitive.

In line with the work of Domanski *et al*.^48^ our results, however, suggest that starting the simulation from bound proteins is more beneficial. Lipids trapped at the dimer interface when the unbound proteins come closer seems to be a severely limiting issue also in US-REMD. Following the walk of the systems in the umbrella space indicates that typically only a few of them actually explore a large range of umbrellas and therefore a large range of protein separations even with the extended simulation time used (Figure S7). Most replicas remain within a restricted range of umbrellas or distances between proteins. The benefit of REMD then relates more to its ability to sort conformations in the umbrella space than to its ability to generate different conformations. This dual aspect of REMD simulation has been described previously for temperature replica exchange simulations^49^. An alternative solution would be to use a mix of bound and unbound configurations of the protein that are more representative of the umbrella potential they start at. But this situation is rapidly reached (a few hundred nanoseconds) in the US-REMD simulation started from bound proteins.

Finally, the convergence of a US-REMD simulation started from bound hSERTs was followed over a 10 μs period (Fig. 3F,G). It is clear that a few μs are needed to remove the memory from the bound state and start collecting relevant information. Similar progression is observed for all the interfaces probed in this study (Figs 3F,G, S8–11). Overall our analysis indicates that the US-REMD simulations are able to reach a reasonable level of convergence within 10 μs. We note that in the present approach we sample only one interface at the time so the proteins do not need to rotate to probe other interfaces. This particularity certainly accelerates the convergence of the simulations.

### The symmetric TM12 dimer is the most stable

**Figure 4:**
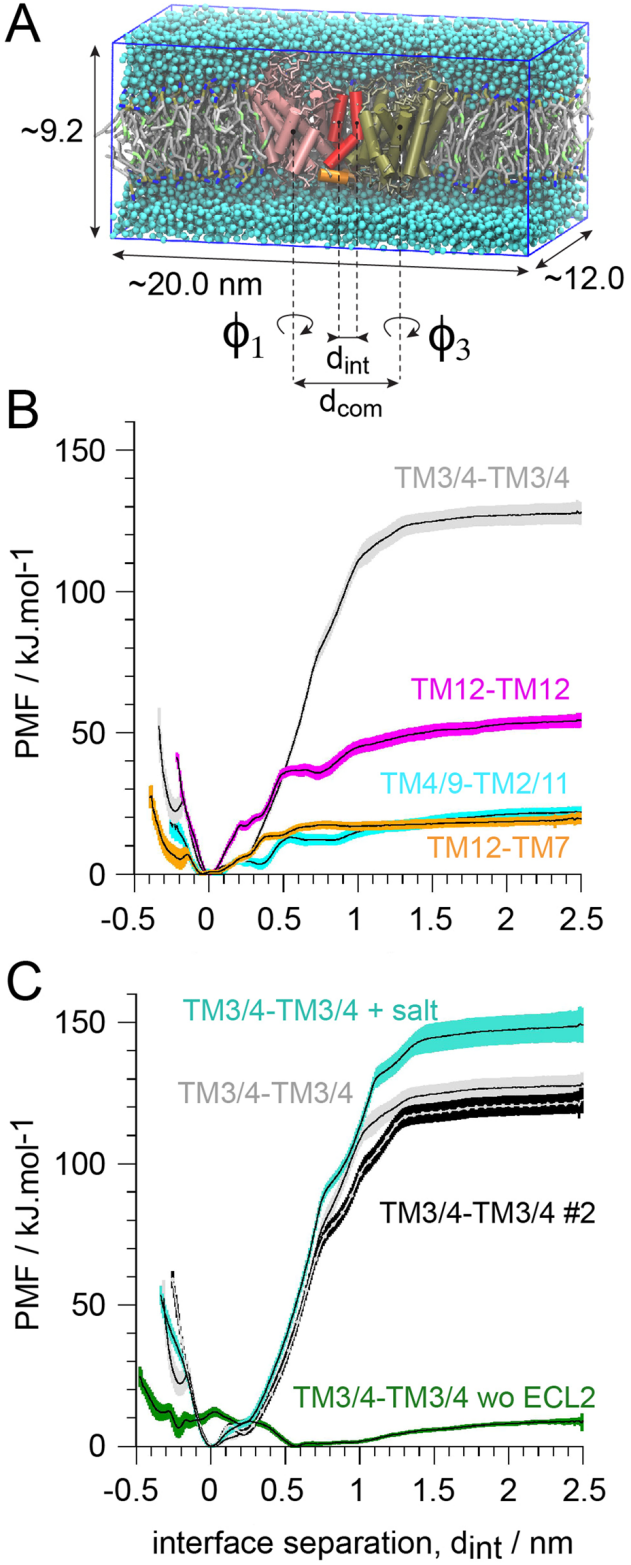
Comparative strength of hSERT dimer interfaces. (**A**) Simulation setup. The transporters transmembrane helices (TM) are shown in tan and pink tubes, but TM11 and TM12 are black and red, respectively, and the C-terminus helix in orange. (**B**) Potentials of mean forces (PMF) of the different hSERT interfaces formed in the self-assembly simulations. (**C**) PMFs of the TM3/4 interface variant: the original TM3/4 and a repeat (#2), in presence of 0.2 M of NaCl (+salt) and with the interaction of the ECL2 between the two monomers removed (wo ECL2).

The small number of binding and unbinding events in the self-assembly simulations (Figures S12, S13) precludes the interpretation of the population of the different interfaces as thermodynamics data^40^. These simulations are typically out of equilibrium. Instead, the relative stability of the four most populated interfaces formed in the self-assembly simulations were assessed by US-REMD simulations that allow the calculation of the PMF of the system as a function of protein separation (Fig. 4). Weak harmonic potentials are used to control the relative orientation of the hSERT monomers and thereby define specific interfaces (Fig. 4A), as done previously^40^.

The depths of the well in the PMF curves suggest that the symmetric interface based on TM3/4 is by far the most stable. Then comes the symmetric TM12 interface, followed by the two asymmetric interfaces, TM4/9-TM2/11 and TM7-12 (Fig. 4). The TM3/4 conformation is stabilized by ~130 kJ/mol, ca. 70 and 110 kJ/mol more than the other interfaces. This represents an unusually high value for protein-protein interactions^2,40,45,48,50–53^. A visual inspection of the umbrella trajectories of this interface, revealed an interaction of the ECL2 (between TM3 and TM4) in the aqueous phase that proved to be difficult to “break” in the simulations and led to an unrealistic conformation where the proteins tilt considerably (Figure S14). The section of the protein interface that spans the membrane hydrophobic interior is solubilized by lipids even in the equilibrated dimer conformation (Figure S14). These observations suggest that the strength of this interface might be the result of the so-called “stickiness” of the MARTINI force field for proteins in the aqueous phase; a known issue only sparsely documented in the literature but a few attempts of improvement have been reported^54^ (D de Yong, X Periole and SJ Marrink, unpublished results).

The involvement of ECL2 in the strength of the TM3/4 interface was further evaluated by determining an additional set of PMFs (Fig. 4C). We first repeated the US-REMD simulation of the TM3/4 interface. The two profiles are within the statistical error of each other. Addition of 0.2 M NaCl to the aqueous phase increased the apparent strength of the interface but the position of the minimum is not affected. Finally, we probed the effect of removing the ECL2-ECL2 interactions between the two hSERTs. The impact is drastic. The stabilization of the interface upon contact simply disappears. This observation was confirmed by the results from a simulation where the proteins were allowed to evolve freely from the dimer conformation without the ECL2-ECL2 interactions being accounted for. The two hSERTs separated within a few microseconds (Figure S15).

Furthermore, we performed simulations of the ECL2-ECL2 starting from an unusual conformation with highly tilted proteins where ECL2 interactions seemed solely responsible for holding the complex together. US-MD and unbiased simulations performed at both atomistic (AT) and coarse grain (CG) resolutions confirmed that the interface is more stable in the CG resolution than in the AT one. More details can be found in S14–17. One aspect that was not explored here is the contribution of applying an elastic network (EN) to preserve the loop structure. An increased rigidity of the loop in the CG resolution is likely to stabilize their interaction. This idea was hinted by the rapid loss of structure in the ECL2 in the AT resolution simulations (Figures S16–19). For the sake of comparison we also ran the TM12-TM12 interface at AT resolution. The interface was found to be stable starting from the dimer conformation derived from the CG simulations. A period of relaxation of the interface lasted for about 100 ns followed by 400 ns of a steady interface (Figure S20).

The strength of the other three interfaces is within a more regular range for membrane protein interactions^53^ (Fig. 4) and do not involve extra-membraneous sections (Fig. 2). The symmetric interface involving TM12 is more stable than the other two with an interaction energy of~55 kJ/mol against ~20 kJ/mol for the other two. The involvement of TM12 at the stronger interface, suggests that it might be the most biologically relevant one, which is fully in line with experimental studies^30,31,43^.

### The interface similar to the LeuT dimer is not accessible to hSERT in a membrane environment

We have also probed the strength of the hSERT dimer interface that would result from an orientation similar to the one found in the crystal structure of LeuT (Fig. 2)^31^. A notable difference in LeuT and hSERT is the location of TM12 relative to the rest of the helical core of hSERT (TM1–11), which results in TM12 being rotated away from the interface when the helical core is positioned as found in the LeuT dimer structure compared to the interface found in the simulations (Fig. 2B). Starting from the separation found in the LeuT crystal structure the two monomers quickly fill the empty gap (~0.6 nm) to form direct contacts between TM9 and TM12 (Figure S21). Strikingly, the prediction of hSERT interface using the web server Cluspro^55^ (with standard weights and using the C2 symmetry option) gave an interface very similar to the LeuT one, with a positional root-man-square distance of 0.31 nm of the backbone Cα. An overlap of the two structures is shown in Figure S21D.

The calculation of the PMF for this interface proved to be impossible with our current methodology due to the shape of the protein interface. The interface resembles two complementary crescent moons (Figs 2, S22). When the distance between the two monomers is increased, a gap forms between the proteins but it is inaccessible to the lipids due to the shape complementarity of the interface and the contacts between TM9 and TM12. Before lipid molecules may enter, the space is field by water molecules (Figure S22A). Once the channel was formed it was stable for distances between the proteins of over 2 nm from the bound conformation. In contrast, in the simulations started from unbound proteins, lipids get and remain trapped at the interface (Figure S22B). Although these observations could argue for a strong interface it was not detected in the self-assembly simulations. The reason for this absence is likely that the proteins need to operate a 20 degrees tilt one relative to the other compared to their equilibrium orientation free in the membrane bilayer (Figure S23). Overall our results suggest that the interface of hSERT corresponding to the one found in the crystal structure of LeuT^31^ or predicted by Cluspro is not accessible in a membrane environment. One can easily imagine that the lack of such environment in the preparation of the crystals and in Cluspro workflow may favor the formation of this interface.

### Effect of phosphatidylinositol bisphosphate (PIP2) on the TM12 interface

**Figure 5:**
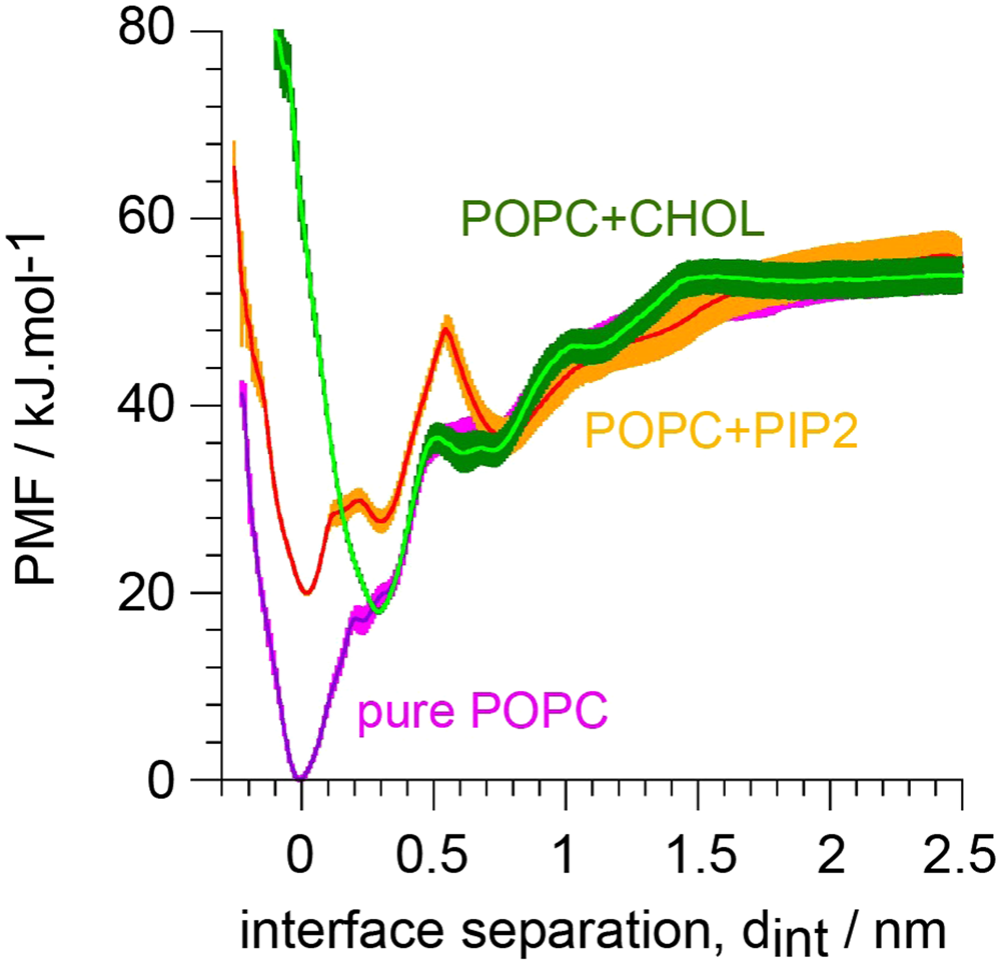
Effect of POPIP2 (C16:0/C18:1-PIP2 lipid) and of cholesterol (CHOL) on the predicted hSERT dimer interface based on the symmetric TM12-TM12 contact. A potential of mean force (PMF) of this interface is shown as determined in a pure POPC membrane (POPC), a mixture POPC:POPIP2 (ratio 9:1, 10%) and a mixture POPC:CHOL (ratio 4:1, 20%).

We have quantified the perturbation of the symmetric TM12-TM12 dimer interface by the lipid 1-palmitoyl-2-oleoyl-PIP2, POPIP2 (Fig. 5). The PMF determined in a POPC:POPIP2 mixture with a 9:1 molecular ratio indicates that the presence of POPIP2 lipids in the membrane decreases the strength of the TM12 hSERT dimer interface in comparison to a pure POPC bilayer. The PMF further reveals that the presence of POPIP2 affects hSERT association in two ways. First, a conformation at an interfacial protein separation, d_int_, of 0.3 nm is stabilized. In the profile from the pure POPC membrane a small bump can be seen at the same d_int_ suggesting that this conformation is also present in pure POPC but only marginally noticeable in the PMF curve. This observation suggests that the presence of POPIP2 alters the balance between the main dimer in POPC (d_int_ = 0 nm) and this alternative conformation at d_int_ = 0.3 nm, so that the minimum at d_int_ = 0 is not as deep in POPIP2 as in pure POPC. Second, an energy barrier forms at d_int_ = 0.55 nm resulting in the stabilization of an additional conformation at d_int_ = 0.75 nm.

**Figure 6:**
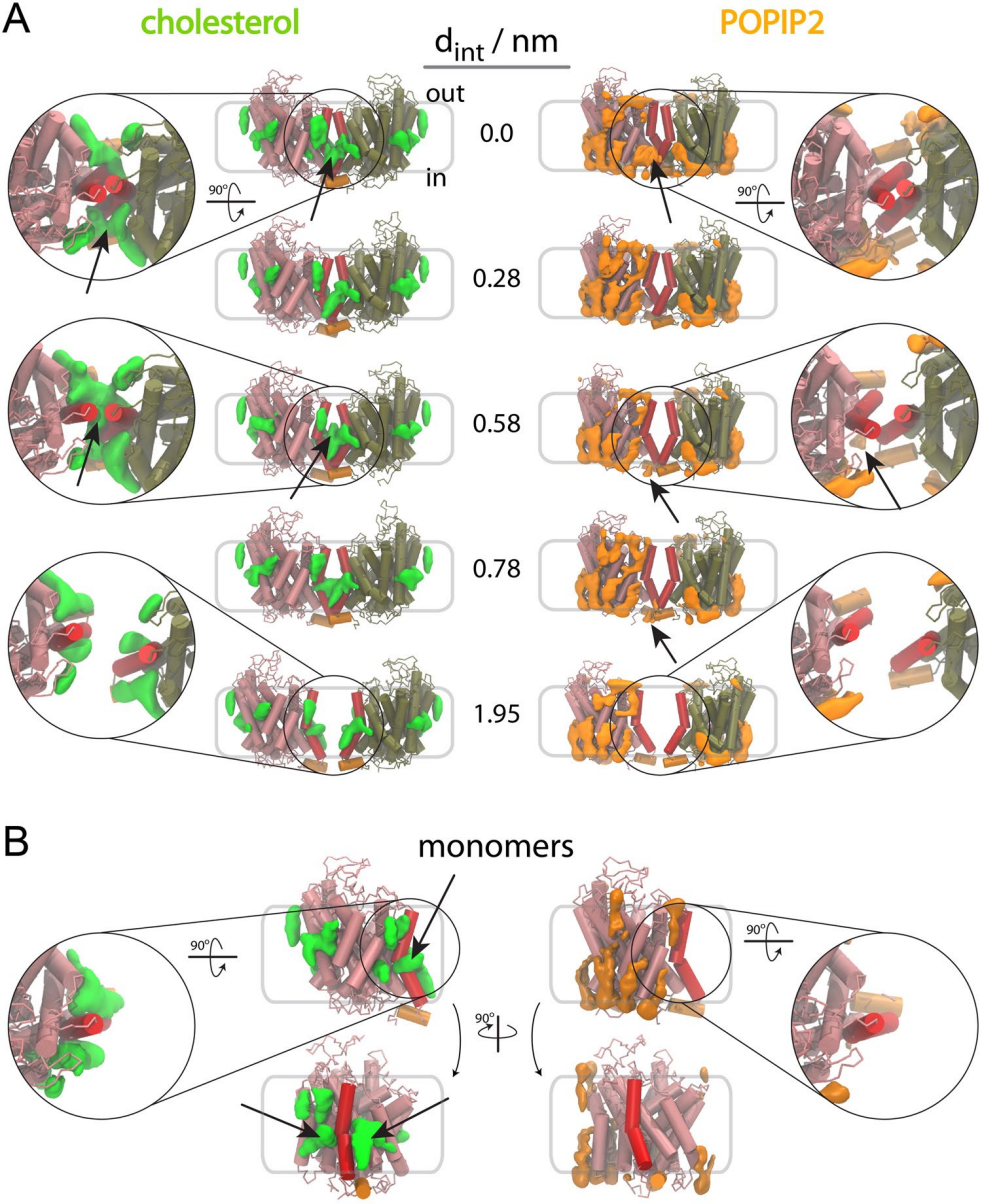
Lipid rearrangement at the dimer interface of the TM12-TM12 hSERT predicted dimer. We show the 3D maps of lipid densities around the hSERT dimers (**A**) and monomer (**B**). The proteins are depicted in tan and pink tubes with TM12 highlighted in red and hSERT C-term helix in orange. Lipid densities are shown in green for cholesterol and orange for POPIP2 (C16:0/C18:1-PIP2 lipid). In (**A**) the densities are shown for five interfacial distances, d_int_, and zoom in of the interface viewed from the extracellular side of the membrane are given for three representative distances. The densities were obtained using the ensemble of conformations found in the umbrella simulation with the reference d_int_ indicated in the figure. Arrows point to relevant densities mentioned in the text. More views are given in Figures S24, 25.

To rationalize the modifications of the PMF triggered by the presence of POPIP2 we calculated the occupational density of POPIP2 in the US-REMD simulations as a function of the distance between the proteins (Fig. 6A). The density maps determined for the monomeric system (Fig. 6B) are similar to the ones measured at different distances between hSERT molecules in the dimer system. There is therefore no straightforward explanation for the effect described above at short distances. However, a slight increase in density close to the interface is observed (indicated by an arrow in Fig. 6A) and could therefore alter the main minimum. Also, a small density observed at d_int_ = 0.58 and 0.78 nm (indicated by an arrow in Fig. 6A) and not observed in the monomer, places partial POPIP2 molecules at the interface between the hSERT monomers and could therefore be responsible for the energy barrier observed at d_int_ = 0.55 nm. A molecule of POPIP2 at the interface could indeed prevent both the association and the separation of the transporters.

### Effect of cholesterol on the TM12 interface

Similarly, we have quantified the effect of CHOL on the symmetric TM12-TM12 dimer interface. The PMF determined in a POPC:CHOL mixture at a 4:1 molecular ratio indicates that, as for POPIP2, the presence of CHOL in the membrane decreases the strength of interaction between hSERTs. In the case of CHOL the perturbation of the PMF occurs mainly at the minimum (d_int_ = 0.0 nm). CHOL does not allow this conformation to form but instead stabilizes an alternative conformation found in POPIP2 at d_int_ = 0.3 nm. The remaining of the PMF curve is similar to the one in pure POPC although CHOL slightly modifies the plateau observed at 0.5–0.7 in pure POPC to form a small energy barrier (Fig. 5).

The 3D maps of the occupational density of CHOL as a function of the distance in our US-REMD simulation were determined to elucidate the effect of CHOL on hSERT interaction as interpreted from the PMF (Fig. 6A). As in the case of POPIP2, the densities of CHOL around each hSERT when the two monomers are far apart are identical to the ones obtained from the monomer simulations. But, in contrast with the case of POPIP2, the densities of CHOL at the interface between the hSERT are different from the monomeric system (Fig. 6B). The change of density is already visible at d_int_ = 0.78 nm (Fig. 6A) and until d_int_ = 0.0 nm. The change in the density maps shows the presence of CHOL right at the interface of the transporters between TM12s, joining CHOL densities from each monomers and on both side of TM12 (Fig. 6A,B). This additional density might relate to the more pronounced shoulder observed at d_int_ = 0.55 nm. However, from the size of the density one could have expected a larger effect on the PMF. At shorter distance, the CHOL density between TM12s disappears to leave only the density on the side of the dimer interface (indicated by an arrow in Fig. 6A). The presence of CHOL at that location is most likely responsible for the lack of a dimer at d_int_ = 0.0 nm.

## Discussion

In the present study we have employed US-REMD simulations with the MARTINI CG model to probe the relative strength of hSERT dimer interfaces predominantly formed during a set of self-assembly simulations. Four interfaces were mainly observed during these simulations (Figs 1,2) and we quantified their relative strength by determining the PMF as a function of the protein separation while their relative orientations are restrained. TM12 abundantly participates to these interfaces and a symmetric arrangement of hSERT primarily involving TM12 is predicted to be the most stable dimer interface probed (Fig. 3). This result is in line with experimental evidence suggesting the involvement of TM12 in the interface of SERT oligomers^30,31,43^.

The predicted conformation of the preferred hSERT dimer resembles the dimer found in the crystal structures of LeuT^31^, a bacterial homologue of MATs. Both the predicted hSERT dimer and the crystal LeuT dimers involve TM12, but in LeuT TM9 equally participates to the interface (Fig. 2). This apparent disparity can be explained by the different position of TM12 relative to the rest of the helical bundle (TM1–11) in hSERT and in LeuT, as well as the kink in TM12 in hSERT. In hSERT, TM12 is distant from TM9 compared to in LeuT (Fig. 2). These findings suggest that TM12 might be an important interaction anchor not only for hSERT and other MATs (DAT and NET) but also for MAT homologues such as LeuT.

The predicted hSERT dimer interface involving TM12 was studied further by quantifying the effects resulting from modifying the bilayer lipid composition that the proteins are embedded in. The PMF were thus determined in POPC:CHOL and POPC:POPIP2 bilayers with 4:1 and 9:1 lipid molecular ratios, respectively, and compared with the case of a pure POPC bilayer (Fig. 5). The effects are strong. Both mixed membranes remove about 20 kJ/mol from the stabilization of the interface upon protein binding in pure POPC. Moreover, in the presence of POPIP2 an energy barrier forms at the distance where the proteins start interacting. However, only a tiny density of POPIP2 was found at the interface between the proteins at that separation. In the case of CHOL, it appeared that the dimer could not reach the same interface as in pure POPC and with POPIP2. In POPC:CHOL, an alternative interface is stabilized that is at ~0.3 nm from the minimum found in pure POPC and in POPC:POPIP2. Extensive densities of CHOL were found at the interface between the proteins that rationalize this effect.

A similar behavior of CHOL was previously reported for G protein-coupled receptors (GPCR). CHOL was suggested to change the relative stability of GPCR interfaces by destabilizing one through a direct interaction to the interface preventing the binding of the proteins^56,57^. Our data is also in line with CHOL affecting integral protein oligomerization by a direct interaction at the protein interface. Note, however, that our data does not exclude a contribution to protein oligomerization by CHOL from affecting bulk mechanical properties of the membrane.

In the case of PIP2, the mechanism of destabilization of the interface is less clear as only residual POPIP2 density is found at the protein interface. Nevertheless, our data suggests that anionic lipids might act on a non-binding surface of proteins by destabilizing that particular interface. This behavior contrasts with earlier reports of anionic lipids having a gluing effect on proteins^32,33^. Cardiolipin (CL), an anionic lipid as POPIP2 but with much bulkier alkyl chains, has been shown to stabilize interactions between complexes of the respiratory chain^32^. We have shown that CL operates by using specific binding sites on both protein partners^33^. This ability of anionic lipid to bind proteins might relate to the observation that PIP2 decreases the exchanges of hSERT molecules in oligomer aggregates^8^. Indeed, we found significant densities of PIP2 at the surface of hSERT (Fig. 6).

Although it is difficult to relate the effect described above in mixed membranes to experimental data, collectively, our results reveal the strong impact of the membrane composition on the strength of integral protein-protein interactions. This is not a new concept. The membrane composition was previously shown to affect the propensity of proteins to oligomerize^35,58,59^ and affect the interface involved^35,56,57^. Here, we present a first quantitative measure of such effects. These observations emphasize the importance of using a native-like membrane environment for such studies and argue in favor of the development of more reliable complex models^60,61^ and new experiments allowing working directly in cells^6^.

The determination of the PMF for the association/dissociation of two full size proteins is a great challenge that we have undertaken earlier using US-MD^40^. Here we have extended our methodology by using US-REMD, a method recently developed^41^ and used on simple systems^48,62^. We found that the benefits of US-REMD simulations are manyfold. First, US-REMD removes the earlier need to combine umbrellas by hand, thereby removing a potential bias. Second, US-REMD increases drastically the sampling at distances where the proteins associate and dissociate; from the distance of contact to ~1 nm. Nevertheless, the US-REMD approach is not the ultimate solution to sampling issues. It could be further improved by coupling it with methods such as Hamiltonian replica exchange, where protein-protein and protein-lipid interactions could be scaled. Third, these two improvements allowed generating reasonably converged PMFs within 10 μs per replica, thus about 300 μs cumulative simulation time. The same level of convergence could in principle be achieved with simple US-MD but with much longer simulation times. Alternatively, one could imagine a manner to combine conventional US simulations in a more automated manner so that the rapid convergence of individual US simulations could be exploited. Filizola and coworkers have combined US-MD with metadynamics to improve the sampling^52^. It is not clear how their approach compares with US-REMD but it allows studying systems in great detail^63^.

In summary, we have benchmarked US-REMD to study full size protein interactions and predicted that the most likely interface of hSERT involves TM12 in a symmetric manner. Furthermore, we have shown that the composition of the embedding membrane strongly affects the strength of the interface. We expect these findings will stimulate new modeling and experimental studies to unravel the precise mechanism behind the effects of lipids reported here. On the modeling side, we showed that US-REMD simulations allow studying protein-protein interactions with a close to atomistic resolution without requiring excessive computer resources. This approach should be easily automated for general use, opening great promise for the prediction of protein-protein interactions in a more native-like environment.

## Methods

### Models and resolutions

An atomistic model for hSERT (residues 74 to 617) was built from its crystal structure bound to paroxetine (PDB ID: 5I6X^27^) to which the following modifications were made: the ligand was removed, the missing side chains were added with Prime v. 3.9 and the protonation state of titrable residues was determined with Propka^64,65^, both as embedded in Maestro (the Schrödinger Suite 2015-1; Schrödinger, LLC); Glu508 was protonated; a disulfide bridge was built between Cys208 and Cys209; Ile291, Thr439 and Tyr110 were mutated back to their native side chain, Ala, Ser and Ala, respectively, using UCSF-Chimera and the Dunbrack rotamer library^66^. Chloride and sodium (placed at the NA1 and NA2 sites) ions and the ligand (serotonin or paroxetine) were not included in the coarse grain model because its resolution (see below) would not reflect their presence.

This atomistic model of hSERT was converted into a MARTINI-v2.2 coarse grain model using the martinize script^67^ and inserted into membrane bilayers using the insane script^68^. The MARTINI force field for lipids^69^, the most recent version of cholesterol^70^ and its extension to proteins^67,71^ were used in combination with the ElNeDyn approach^72^ to maintain the secondary and tertiary structure of the protein. This CGMD approach is well suited to study complex biological membranes^53,61,73,74^.

The atomistic hSERT model was also simulated at atomistic resolution. A monomer was simulated to quickly relax its structure. Dimeric conformations were extracted from the CG simulations and back-mapped to explore their stability at atomistic resolution. We used the script *backwards* to backmap CG structures^75^. The CHARMM36 force field^76–78^ was used to describe the protein, the lipids and the aqueous phase. Internal Cl^−^ and Na^+^ ions, and water molecules found in the crystal structure (PDB ID: 5I6X^27^) were included in the model but not the ligand.

### Simulations

All molecular dynamics (MD) simulations were performed using the GROMACS simulation package version 5.1^79^. Conventional simulation setups associated with the MARTINI^67,80,81^ and CHARMM force fields^76–78^ were used. For the CG simulations, these include a 20 fs time step for production run, a 0.9 nm cutoff and a 500 kJ/mol/nm^2^ force constant for ElNeDyn. The non-bonded interactions (van der Waals and electrostatic) received a cutoff of 1.1 nm and were shifted to zero using the potential-modifier implementation in GROMACS. A relative dielectric screening constant of 15 and the Verlet neighbor search^82^ were used. The protein (hSERT), the membrane bilayer (POPC) and the aqueous phase (water plus ions when present) were coupled independently to an external temperature bath at 300 K using a Berendsen^83^ and Bussi^84^ thermostats (τ_T_ = 0.5 ps) for equilibration and for production, respectively. The pressure was weakly coupled^83^ to an external bath at 1 bar using a relaxation time of 2 ps following a semi-isotropic pressure scheme. A list of the simulations performed is given as supplementary information (Table S1). Most analysis were performed using frames saved every 1 ns.

In the atomistic simulations a 2 fs time step was used together with the LINCs algorithm^85^ to constrain the bonds involving hydrogens. Both temperature and pressure were weakly coupled to 310 K and 1 atm using the Berendsen approach^83^. The decomposition of the system for temperature and pressure control was as for the CG resolution. The non-bonded interactions received a 1.2 nm cutoff and the electrostatics were treated using PME at longer distances, while the force-switch potential modifier was used for the vdW interactions^79^.

### Potential of mean force

To calculate the potential of mean force (PMF) between two hSERTs we used our combination of virtual bond algorithm^86^ (VBA, Figure S26) with a slightly improved umbrella sampling approach (US)^40^. VBA is used to hold the relative orientation of the two proteins so that a particular interface is probed. In addition to the conventional US method, we have used the approach that combines US with replica exchange MD^41^. This straightforward implementation takes advantage of the exchange of replicas between the umbrella windows to accelerate sampling convergence over the whole range of the reaction coordinate^48,62,87^. The umbrella trajectories were unbiased and combined using a WHAM^88,89^ implementation that takes into account the use of VBA^40^. The equilibrium distance of each umbrella potentials were first defined every 0.1 nm. Smaller intervals were used when test simulations (500 ns) indicated a low exchange (<10%) between consecutive umbrellas. The rates of exchange varied between 0.15 and 0.40 and the production runs were 10 to 18 μs (Table S1). The final PMF and the associated statistical errors were obtained from the average of 100 PMF curves generated by a bootstrap procedure where half-sized umbrella windows were used for resampling.

### Systems

Two different setups for the CG self-assembly simulations were used. A system with sixteen hSERT monomers was run in 10 repeats each lasting 30 μs and a system consisting of 64 hSERT monomers was run for 250 μs. The small setup was started with the sixteen copies of hSERT placed on a four-by-four grid using a pre-equilibrated system containing one hSERT in a membrane patch made of 99 1-palmitoyl-2-oleoyl-sn-glycero-3-phosphocholine (POPC) lipid molecules (Fig. 1). A 500 ns simulation with soft position restrain (100 kJ/mol/nm^2^ in the membrane plane) on a central residue of hSERT (Leu338) was used to randomize the orientation of the sixteen copies. The large system with 64 hSERT monomers was prepared in a similar s.

For the calculations of the potential of mean force we used systems composed of a pair of hSERT molecules inserted into a rectangular box (Fig. 2A) containing ~630 POPC molecules, or a mixture of 572 POPC and 63 1-palmitoyl-2-oleoyl-PIP2 (POPIP2) molecules (9:1 ratio) and a mixture of 507 POPC and 126 CHOL molecules (4:1 ratio). The pairs of hSERTs were dimer conformations extracted from the self-assembly simulations, which were relaxed in the corresponding lipid mixture prior to US simulations. The orientation of the hSERT dimers was chosen such that their distance (the reaction coordinate) would vary along the long side of the box. A position restrain (10 kJ/mol/nm^2^) was applied on each hSERT molecule (backbone bead of Leu338) such that they remain close to the center of the small side of the box.

The systems studied at the atomistic resolution contained a monomeric structure of hSERT embedded into a 380 POPC lipid bilayer and two dimer conformations back-mapped from CG structures used in the calculation of the PMFs. These systems were neutralized by adding 0.2 M NaCl in the aqueous phase.

### Cluster analysis

Clustering of the hSERT dimers formed in the self-assembly simulations was performed as previously described^40^. Briefly, dimer conformations with a center-of-mass separation less than 5.3 nm were collected from the 10 simulations containing sixteen hSERTs and concatenated into a single file. Clusters were constructed using the GROMOS approach^90^ using a similarity cutoff of 0.4 nm. Modifying the cutoff value changed the relative population of the clusters but not their presence and only slightly their order.

### Limitations of the methodology

Although, overall, our CGMD methodology and, in particular, the determination of protein-protein interaction strength have been very successful in bringing to light new principles of membrane proteins and their interactions^42,53,61,73,74^, it is important to point out some of the limitations underlying our model and methodology. First, the processes studied involve slow motion of proteins and lipids. Although we have significantly improved our methodology by using US-REMD instead of US-MD simulation as in our previous work, and increased the total simulation time used to generate a PMF by more than a factor of 10, most processes are still under-sampled in our simulations. The lack of sampling is especially true in the self-assembly simulations. Moreover, the US-REMD approach is not a final solution to interface sampling. It still shows signs of sampling limitation and could be combined with alternative enhancing methods. A second limitation of our approach might be the use of a CG representation of the interactions in the system. A known issue for protein-protein interactions modeled by the MARTINI coarse grain is the apparent over-estimation of their strength in the aqueous phase^54^. We have shown here using a series of CG and AT simulations that the symmetric interface TM3/4-TM3/4 is unrealistically stabilized by loop-loop interactions in the extracellular side of the membrane. There is however no evidence that interactions between full size protein are over-stabilized by the MARTINI force field in a membrane environment. Although it might seem the case from a recent study of simple transmembrane helices in a lipid bilayer^91^, protein/protein and protein/lipid interactions are most likely not additive. More studies will be required to reveal the delicate balances of the forces involved. The model of POPIP2 also showed unexpected collective behavior. We observed arrays of a few molecules interacting through the stacking of their rings. Considerable efforts have been and are being put into fixing these issues. The next version of the MARTINI FF should provide remedies to most of theses limitations.

## Electronic supplementary material

Supplementary material
