# Supplementary material for: Dimer Interface of the Human Serotonin Transporter and Effect of the Membrane Composition
Source: Sci Rep. 2018 Mar 23;8:5080. doi: 10.1038/s41598-018-22912-7 (PMC5865177; doi:10.1038/s41598-018-22912-7)
Supplement: Supplementary file 1 — Supplementary material [file 41598_2018_22912_MOESM1_ESM.pdf]

Supplementary material for:

Dimer Interface of the Human Serotonin Transporter and Effect of the Membrane Composition

Xavier Periole*, Talia Zeppelin and Birgit Schiøtt*

Interdisciplinary Nanoscience Center, Department of Chemistry, Aarhus University, Aarhus, Denmark.

Email: x.periole@chem.au.dk, birgit@chem.au.dk

Additional discussion on TM3/4 interface.
To further evaluate the strength of this TM3/4 interface we extracted a conformation from a US-REMD CG simulation with a large tilt of the proteins and contact between the extracellular loops 2 (ECL2). This conformation was back-mapped to an atomistic resolution and simulated at both AT and CG resolutions. Two simulations used the US setup at the distances of 7.0 and 7.2 nm. In a third simulation the proteins were left free to evolve. The detailed analysis of the simulations (see Figure S14-17) clearly support that the interactions involving ECL2 stabilize this dimer conformation in the CG much more in the CG than at the AT resolution. Contacts in the atomistic systems were rapidly lost, while consolidated in the CG ones. Although we cannot rule out that this conformation is biologically relevant to the system its strength is certainly overestimated in the CG simulations as the result of the presence of a significant interface in the aqueous face. There has also not been any report of this interface (TM3/4) for any MAT or homologues.

Table S1. Simulations performed.

System	membrane composition	time simulated	starting conformation	exchange trial/ps	hSERT interface	note
**Coarse Grain resolution**						
**Reference simulations**						
1 hSERT	POPC	10 μs				
1 hSERT	POPC:CHOL	10 μs				
1 hSERT	POPC:PIP2	10 μs				
time simulated		30 μs				
**Self assembly simulations**						
16 hSERTs	POPC	10 x 30 μs				
64 hSERTs	POPC	250 μs				
**US-MD simulations**						
2 hSERTs	POPC	31 x 1.1 μs	proteins bound		TM12-TM12	
2 hSERTs	POPC	31 x 1.1 μs	proteins apart		TM12-TM12	
2 hSERTs	POPC	31 x 1.1 μs	proteins bound		TM12-TM7	
2 hSERTs	POPC	31 x 1.1 μs	proteins apart		TM12-TM7	
2 hSERTs	POPC	31 x 1.1 μs	proteins bound		hSERT on LeuT	
2 hSERTs	POPC	31 x 1.1 μs	proteins apart		hSERT on LeuT	
total		**204.6 μs**				
**US-REMD simulations**						
tests						
2 hSERTs	POPC	31 x 0.9 μs	proteins bound	20	TM12-TM12	
2 hSERTs	POPC	31 x 2.6 μs	“	200	TM12-TM12	
2 hSERTs	POPC	31 x 0.9 μs	“	2000	TM12-TM12	
2 hSERTs	POPC	31 x 2.6 μs	proteins apart	200	TM12-TM12	
production						
2 hSERTs	POPC	31 x 10 μs	proteins bound	20	TM12-TM12	
2 hSERTs	POPC	31 x 10 μs	proteins bound	20	TM12-TM7	
2 hSERTs	POPC	31 x 10 μs	proteins bound	20	TM4/9-TM2/11	
2 hSERTs	POPC	31 x 10 μs	proteins bound	20	TM3/4-TM3/4	
2 hSERTs	POPC	31 x 0.5 μs	proteins bound	20	hSERT on LeuT	
2 hSERTs	POPC:CHOL	31 x 10 μs	proteins bound	20	TM12-TM12	
2 hSERTs	POPC:PIP2	31 x 18 μs	proteins bound	20	TM12-TM12	
total		**2340.5 μs**				
**Simulations for the TM3/4 interface**						
US-REMD						
2 hSERTs	POPC	31 x 10 μs	proteins bound	20	TM3/4-TM3/4	same again
2 hSERTs	POPC	31 x 10 μs	proteins bound	20	TM3/4-TM3/4	0.2 M salt
2 hSERTs	POPC	31 x 10 μs	proteins bound	20	TM3/4-TM3/4	ECL2 off
total		**930 μs**				
MD						
2 hSERTs	POPC	5 μs	proteins bound		TM3/4-TM3/4	unbiased
US-MD						
2 hSERTs	POPC	2 μs	proteins bound		TM3/4-TM3/4	US at 7.0 nm
2 hSERTs	POPC	2 μs	proteins bound		TM3/4-TM3/4	US at 7.2 nm
**Atomistic resolution**						
MD						
2 hSERTs	POPC	1 μs	proteins bound		TM3/4-TM3/4	unbiased MD
US-MD						
2 hSERTs	POPC	0.5 μs	proteins bound		TM3/4-TM3/4	US at 7.0 nm
2 hSERTs	POPC	0.5 μs	proteins bound		TM3/4-TM3/4	US at 7.2 nm

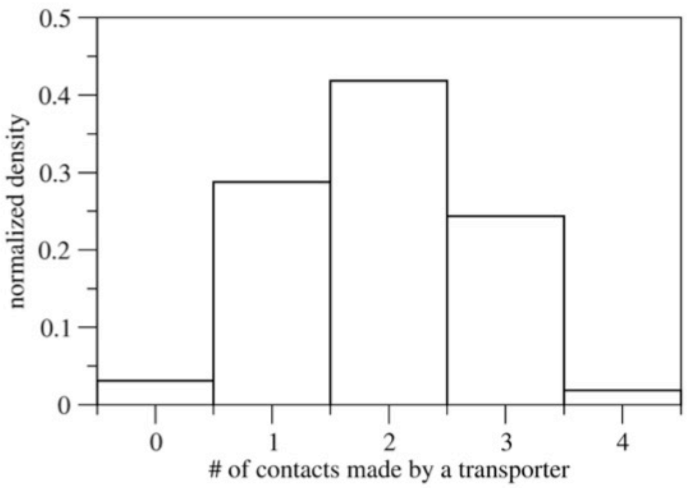

Figure S1. Distribution of the number of contacts made by a transporter. The data was collected over the ten final conformations (30 μs) of the self-assembly simulations containing sixteen copies of the protein. Two proteins were considered in contact if they shared more than 5 contacts (in order to remove transient contacts, see Figure S7 and S12-13) within 0.7 nm.

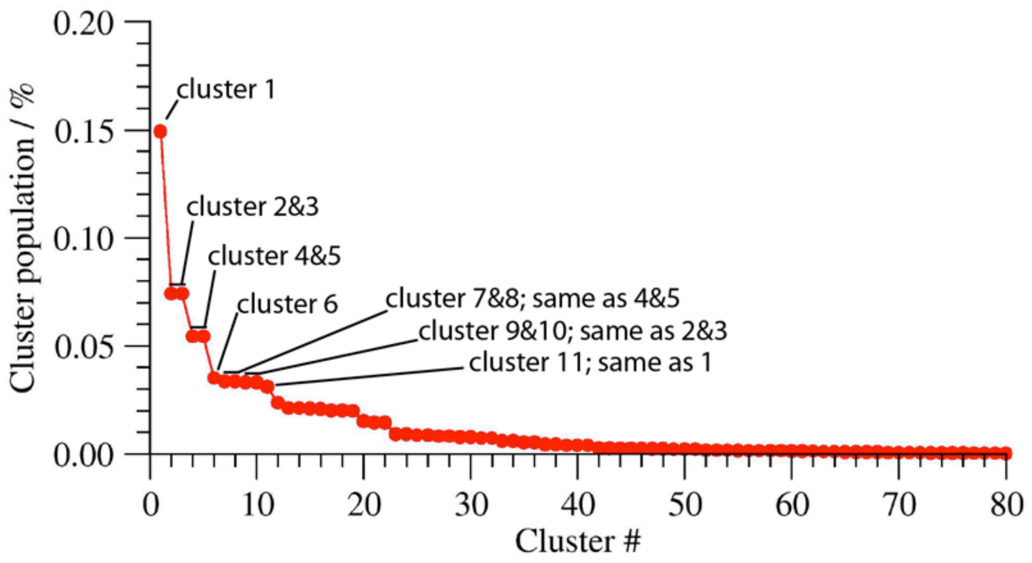

Figure S2. Cluster population of the hSERT dimer conformations extracted from 10 self-assembly simulations. Only conformations with a distance less than 5.3 nm between the centers-of-mass of the two proteins were used for the cluster analysis. A root-mean-square-distance of 0.4 nm was used to define similarity. The apparent repeat of the clusters is due to the symmetry of dimers that do not hold a C2 axis and therefore cannot be superimposed e.g. clusters 2/3 and 4/5, which are therefore in practice combined.

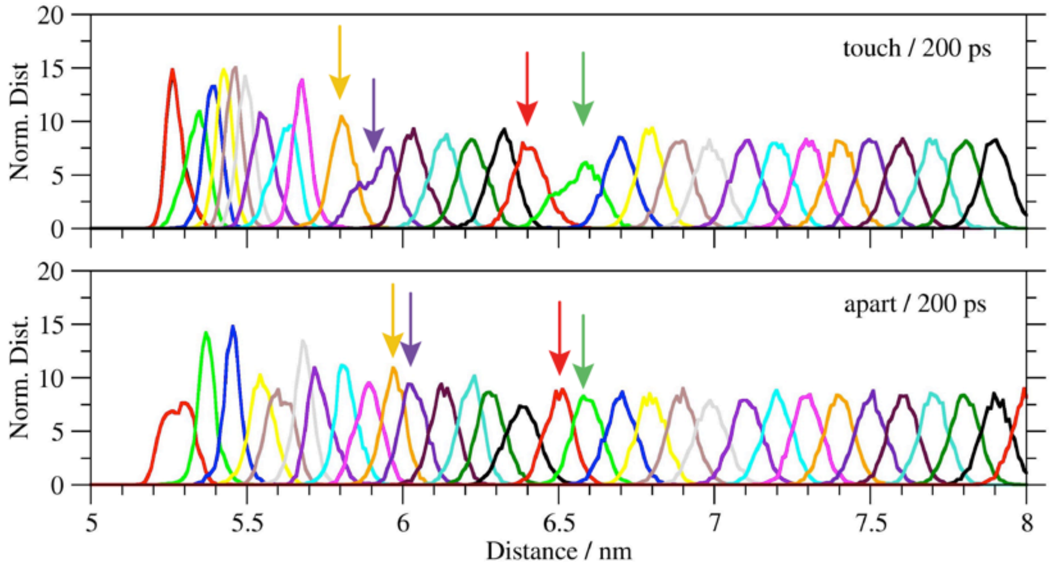

Figure S3. Histograms determined from US-MD started with proteins bound (touch) and separated (apart). The time interval between exchange trials was 200 ps in these simulations. Histograms with the same reference value for the umbrella potential use the same color. One can appreciate how the histograms at long distance are identical but as the distance decreases they start to diverge at 6.4 nm e.g. there is a much higher density of histograms at 5.4-5.5 in the touch simulations while similar density appears around 6 nm in the apart simulation. This difference reflects the difficulty to separate and associate for the proteins in the touch and apart simulations, respectively. Some differences are highlighted by arrows.

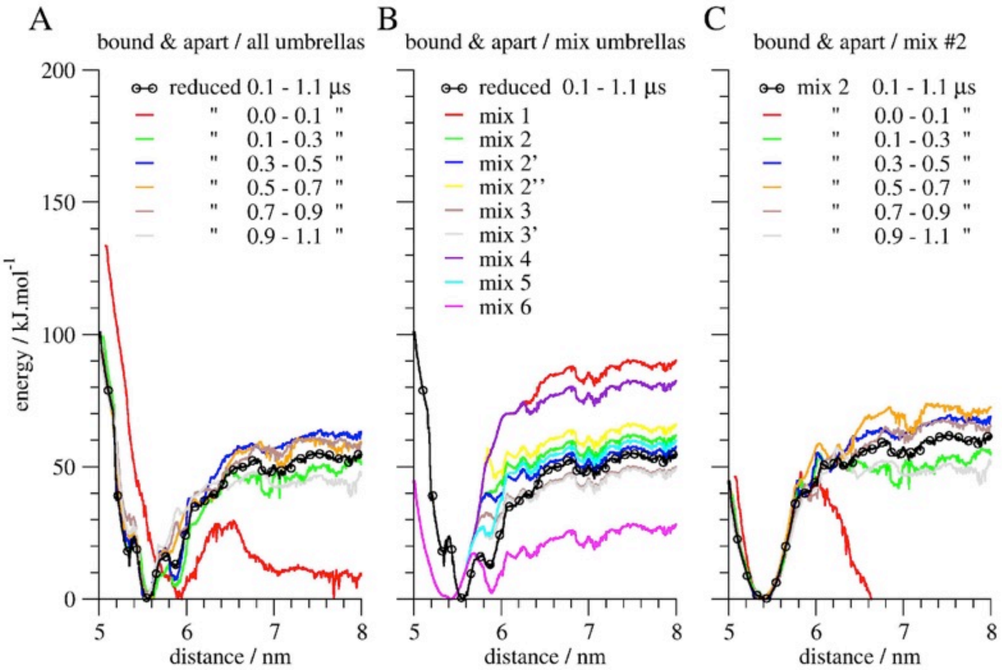

D

Distance nm		50	51	52	53	54	55	56	57	58	59	60	61	62	63	64	65	66	67	68	69	70	71	72	73	74	75	76	77	78	79	80
full	bound	x	x	x	x	x	x	x	x	x	x	x	x	x	x	x	x	x	x	x	x	x	x	x	x	x	x	x	x	x	x	x
	apart	x	x	x	x	x	x	x	x	x	x	x	x	x	x	x	x	x	x	x	x	x	x	x	x	x	x	x	x	x	x	x
reduced	bound	x	x	x	x	x	x	x	x	x	x	x	x	x	x	x	x															
	apart	x	x	x	x	x	x	x	x	x	x	x	x	x	x	x	x	x	x	x	x	x	x	x	x	x	x	x	x	x	x	x
mix 1	bound	x	x	x	x	x	x	x	x	x	x	x	x	x	x	x	x															
	apart												x	x	x	x	x	x	x	x	x	x	x	x	x	x	x	x	x	x	x	x
mix 4	bound	x	x	x	x	x	x	x	x	x	x	x	x	x																		
	apart												x	x	x	x	x	x	x	x	x	x	x	x	x	x	x	x	x	x	x	x
mix 2	bound	x	x	x	x	x	x	x	x	x	x	x																				
	apart										x	x	x	x	x	x	x	x	x	x	x	x	x	x	x	x	x	x	x	x	x	x
mix 2'	bound	x	x	x	x	x	x	x	x	x	x																					
	apart										x	x	x	x	x	x	x	x	x	x	x	x	x	x	x	x	x	x	x	x	x	x
mix 2"	bound	x	x	x	x	x	x	x	x	x	x																					
	apart											x	x	x	x	x	x	x	x	x	x	x	x	x	x	x	x	x	x	x	x	x
mix 3	bound	x	x	x	x	x	x	x	x	x																						
	apart										x	x	x	x	x	x	x	x	x	x	x	x	x	x	x	x	x	x	x	x	x	x
mix 3'	bound	x	x	x	x	x	x	x	x	x	x																					
	apart									x	x	x	x	x	x	x	x	x	x	x	x	x	x	x	x	x	x	x	x	x	x	x
mix 6	bound	x	x	x	x	x	x	x	x	x																						
	apart								x	x	x	x	x	x	x	x	x	x	x	x	x	x	x	x	x	x	x	x	x	x	x	x
mix 5	bound	x	x	x	x	x	x	x	x	x	x	x	x	x																		
	apart									x	x	x	x	x	x	x	x	x	x	x	x	x	x	x	x	x	x	x	x	x	x	x

Figure S4. Analysis of efficiency and convergence of regular US-MD as we used previously (see reference 40 in the main manuscript). Simulations performed and used for analysis were started from either proteins being in contact (touch) or separated (apart). A) Convergence of a US simulation including all windows touch and apart. B) Illustration of the effect of feeding WHAM with only a selected set of umbrella simulations; typically aiming at removing windows showing a clear unrealistic behavior, which is often the case when starting with proteins apart, lipid molecules get stuck in between the proteins. One can appreciate the improved resolution of the bound state when the umbrella simulations starting from proteins apart are removed from the set of simulations used. In the present case we found that the mix 2 is the best compromise as the umbrella simulations started from the bound and the unbound configurations of the proteins overlap smoothly and do not affect each other. C) Convergence analysis of a US-MD simulation with a given set (mix 2) of umbrella simulations considered. Time windows of 0.2 µs were used except for the first window in which case 0.1 µs was used. D) Set of umbrella simulations considered. An umbrella simulation was positioned at each 0.1 nm from 5.0 to 8.0 nm. A cross indicates that the umbrella was included and the light green and orange shadings highlight inclusion and overlap, respectively, of bound and unbound windows.

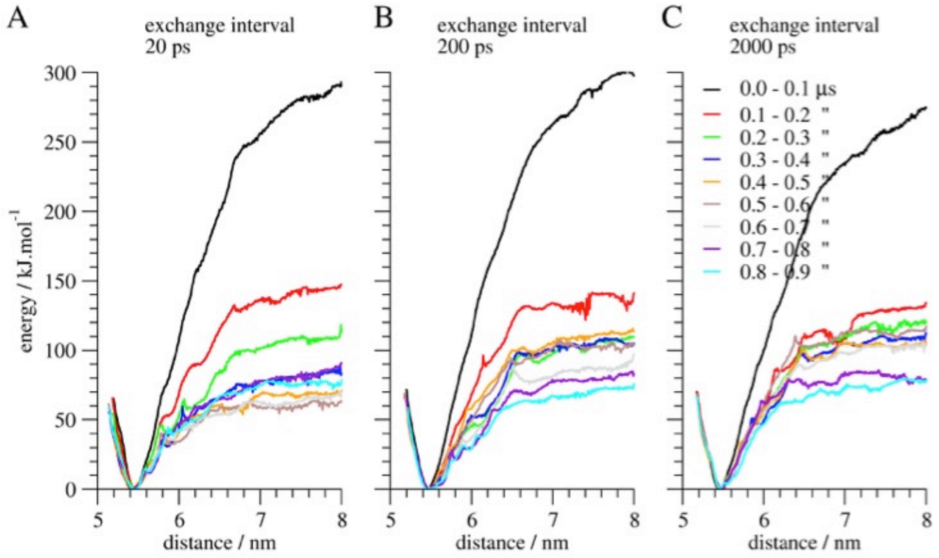

Figure S5. Efficiency of US-REMD simulation as a function of exchange interval between exchange trials. Three values of intervals were used: A) 20, B) 200 and C) 2000 ps. These simulations were started from conformations where the proteins are in contact. The potentials of mean force are plotted as a function of the simulation time over a period of 0.9 μs with a 0.1 μs time window. Exchange trials every 20 ps are overall more effective as once can judge by the rapid convergence of the curves with the simulation time. In the cases of attempt of exchange every 200 and 2000 ps, one can appreciate that while the bound state is explored similarly by the three setups, the simulations using intervals of 200 and 2000 ps take more time to lower their level at medium (~6 nm) and long distances (>6.5 nm). Note that only the simulation using a 20-ps interval seems to have reached convergence, but is not actually converged. See Figure 3 in the main text for more details. The curves were aligned at the minimum. Simulations were performed on the interface 1 involving TM12 and presented in the main manuscript.

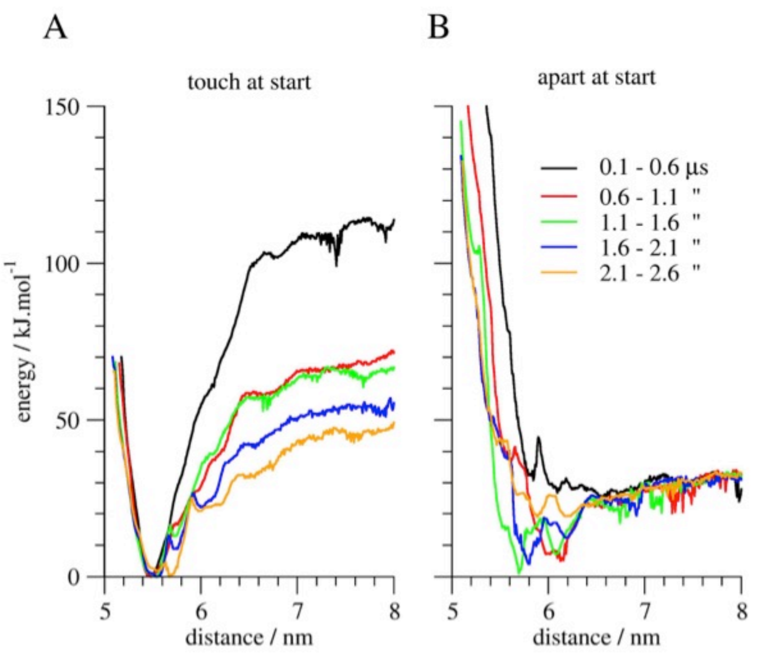

Figure S6. Comparison of US-REMD simulations with starting structure where the proteins are A) bound (touch at start) and B) unbound (apart at start). These simulations use a 200 ps time interval between exchange trial and ran for ~2.6 µs. The progress of the simulations is shown for windows of 0.5 µs. It is clear that the simulation starting with the proteins apart has difficulties to reach the same level of sampling at short distances. Simulations were performed on the interface 1 involving TM12 and presented in the main manuscript.

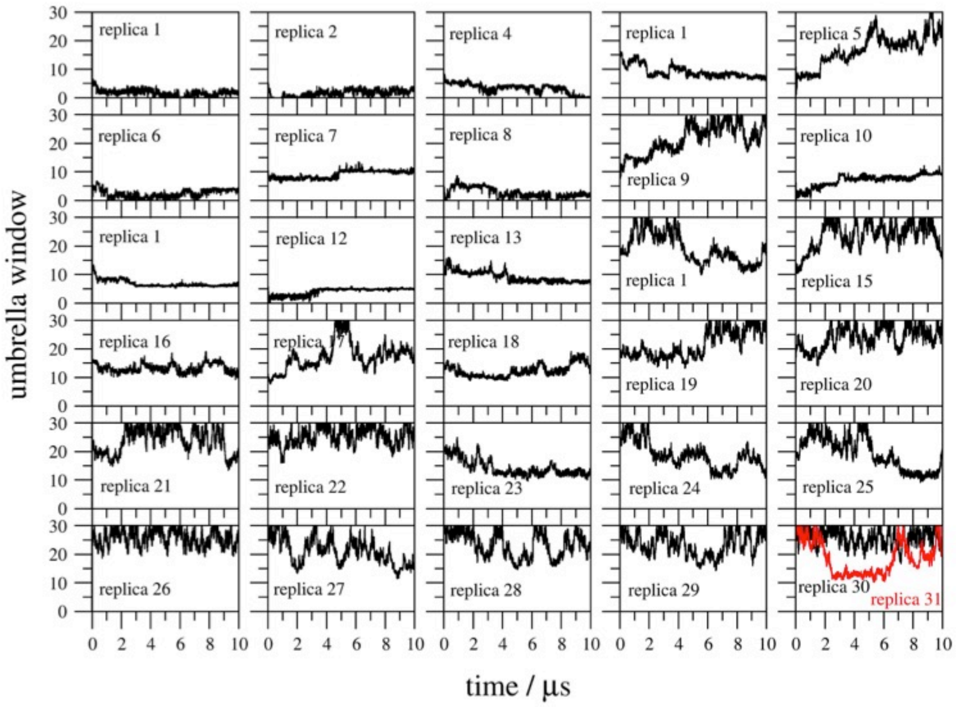

Figure S7. Walk of replicas over the different umbrella widows. The position of the replica in the 31 umbrellas is shown a running average over 100 points saved every 20 ps. The last two replicas, 30 and 31, are shown in the last graph. The US-REMD simulation of cluster 1 (symmetric TM12 interface) started from bound proteins and using a 20 ps interval between exchange trials was used.

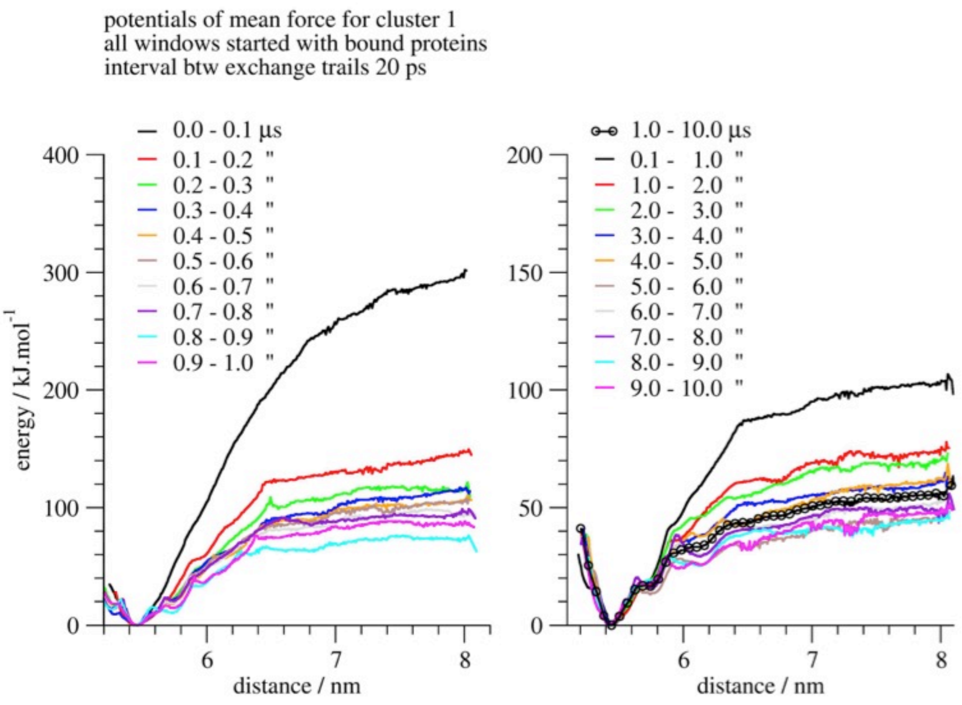

Figure S8-1. Convergence of the US-REMD simulation using a 20 ps interval when starting from configurations of the proteins bound for cluster 1.

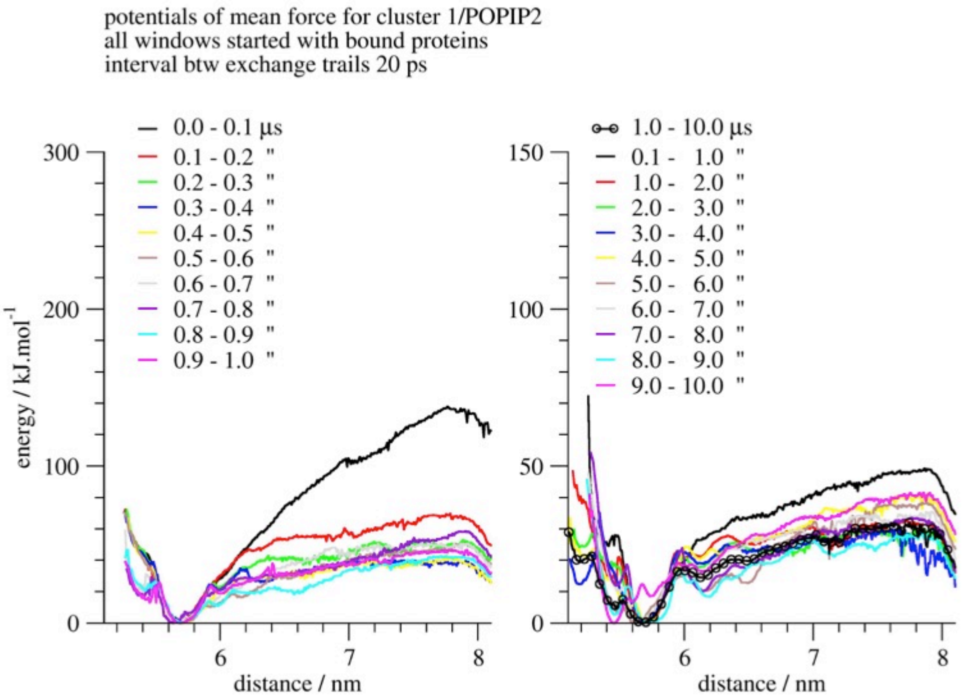

Figure S8-2. Convergence of the US-REMD simulation using a 20 ps interval when starting from configurations of the proteins bound for cluster 1 embedded in a membrane bilayer of mixed POPC:POPIP2 with a 9:1 molecular ratio.

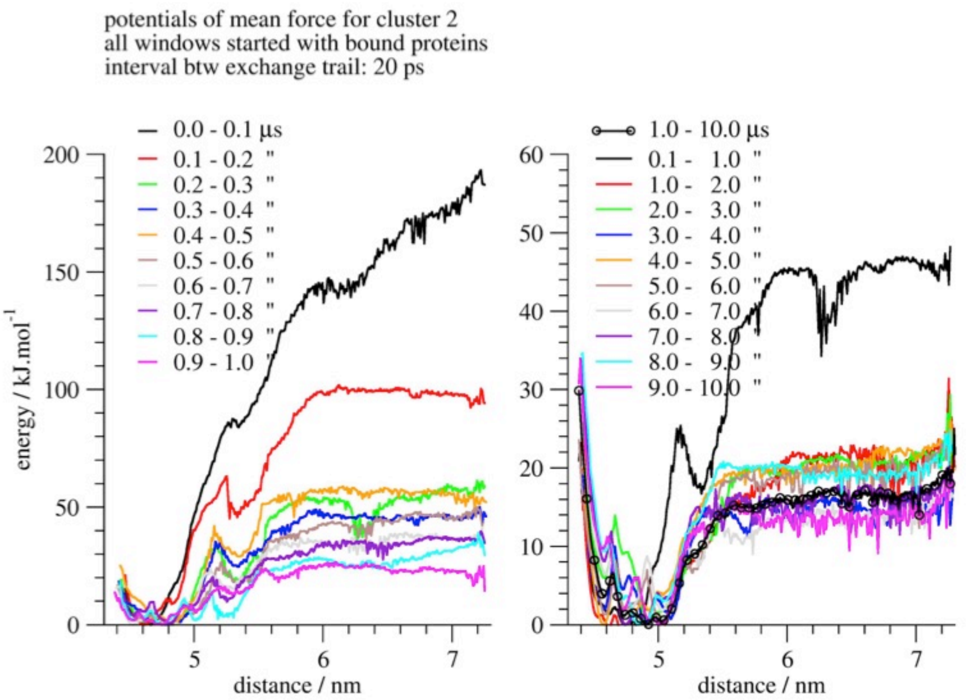

Figure S9. Convergence of the US-REMD simulation using a 20 ps interval when starting from configurations of the proteins bound for cluster 2.

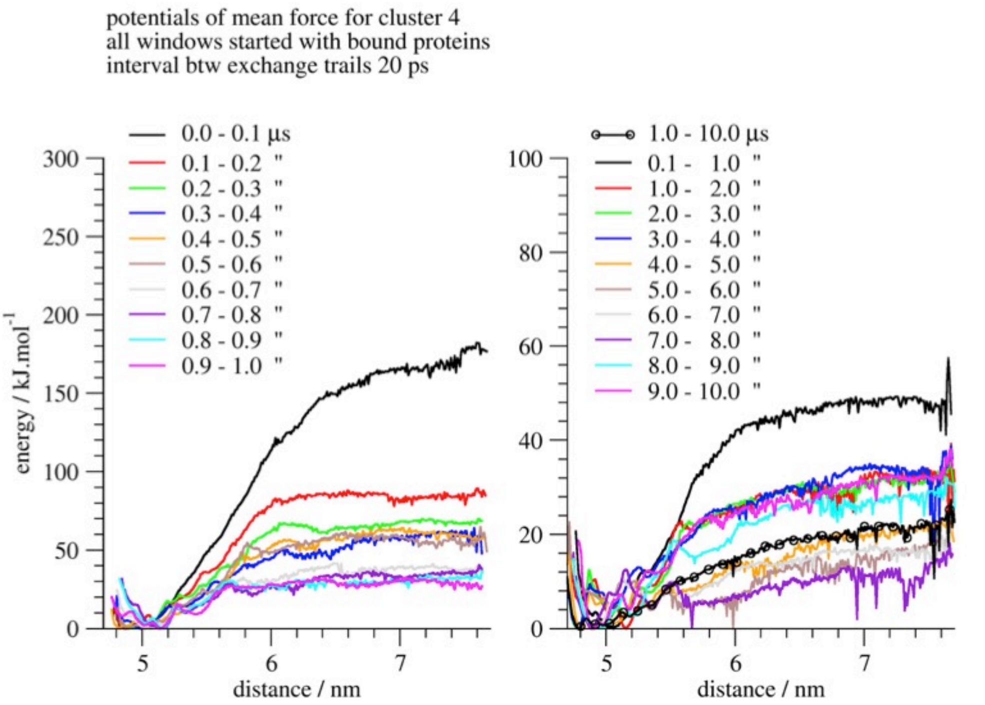

Figure S10. Convergence of the US-REMD simulation using a 20 ps interval when starting from configurations of the proteins bound for cluster 4.

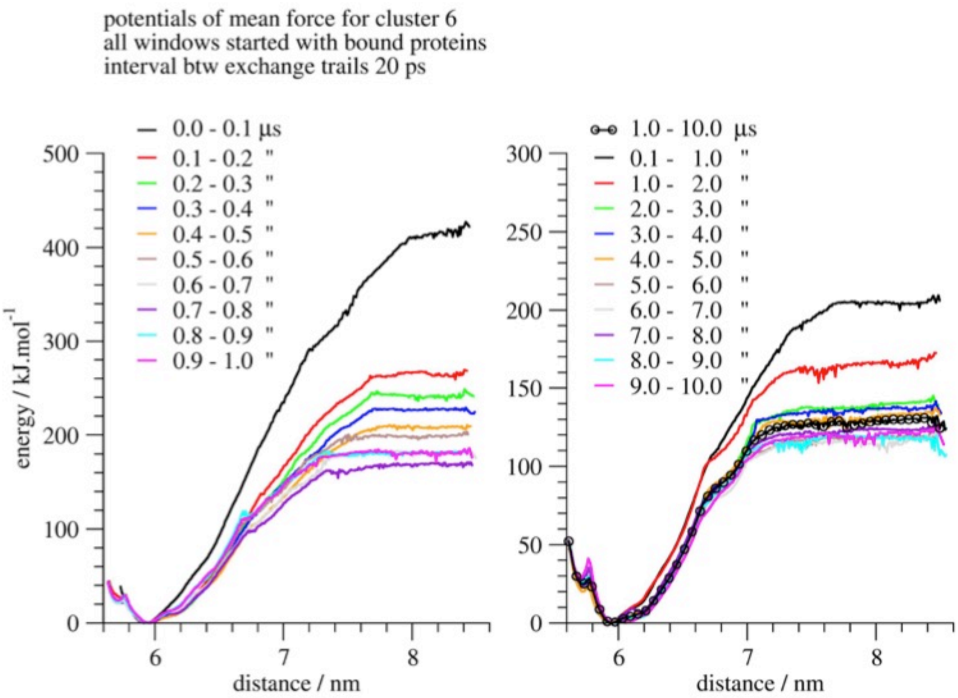

Figure S11-1. Convergence of the US-REMD simulation using a 20 ps interval when starting from configurations of the proteins bound for cluster 6.

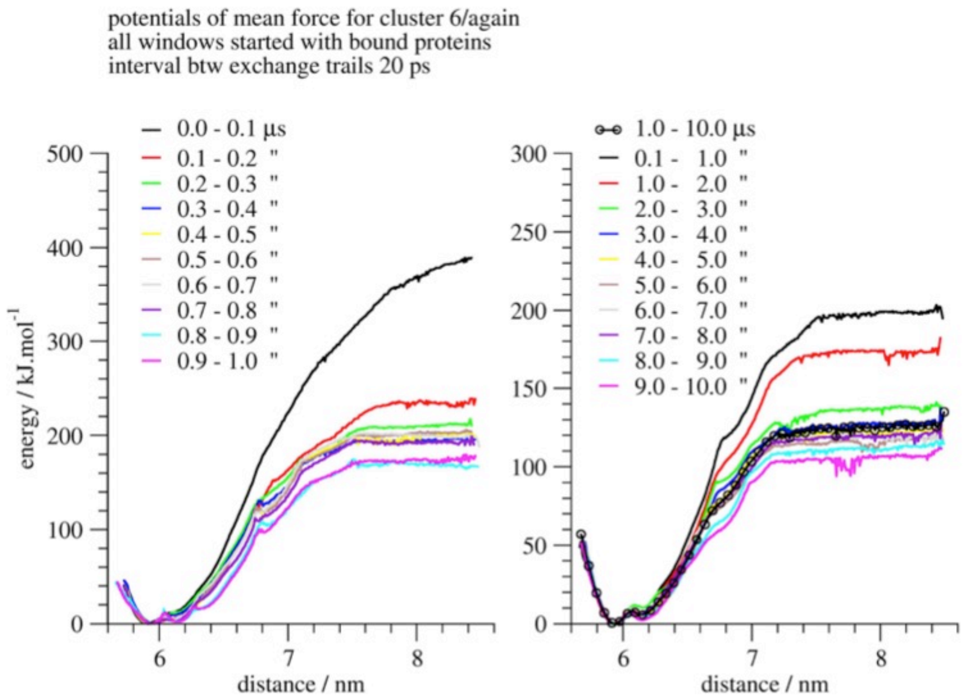

Figure S11-2. Convergence of the US-REMD simulation using a 20 ps interval when starting from configurations of the proteins bound for cluster 6, simulation #2.

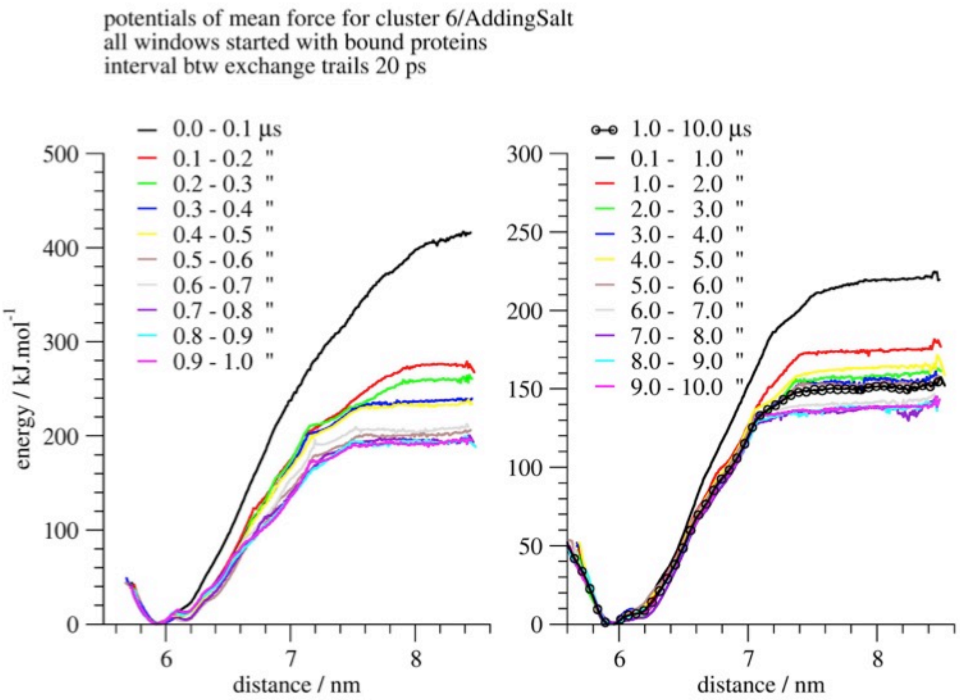

Figure S11-3. Convergence of the US-REMD simulation using a 20 ps interval when starting from configurations of the proteins bound for cluster 6 with 0.2 M NaCl.

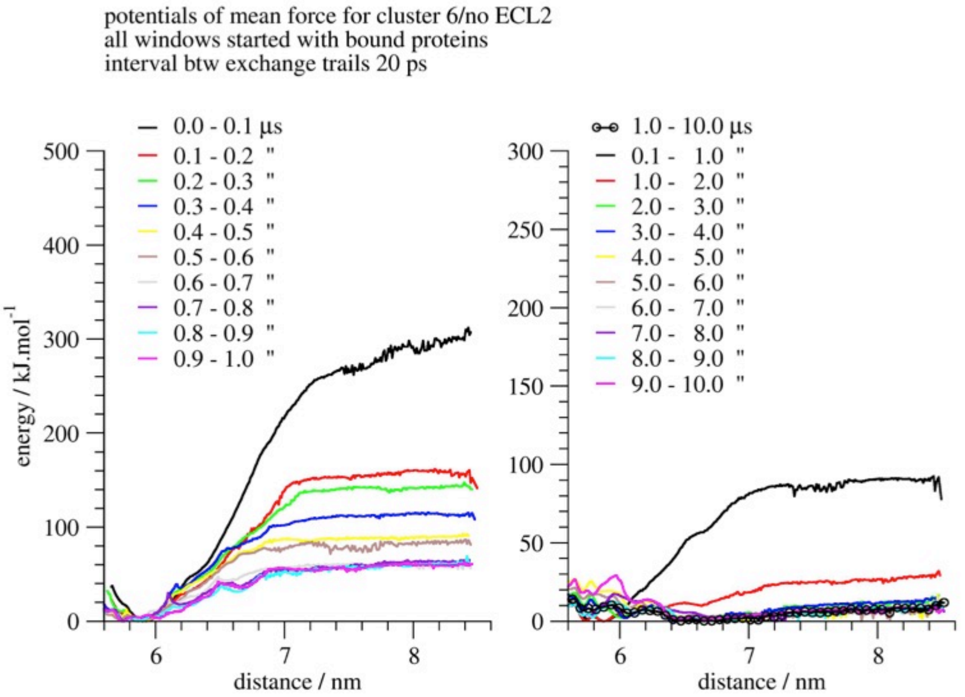

Figure S11-4. Convergence of the US-REMD simulation using a 20 ps interval between starting from configurations of the proteins bound for cluster 6 with the ECL2/ECL2 interactions removed.

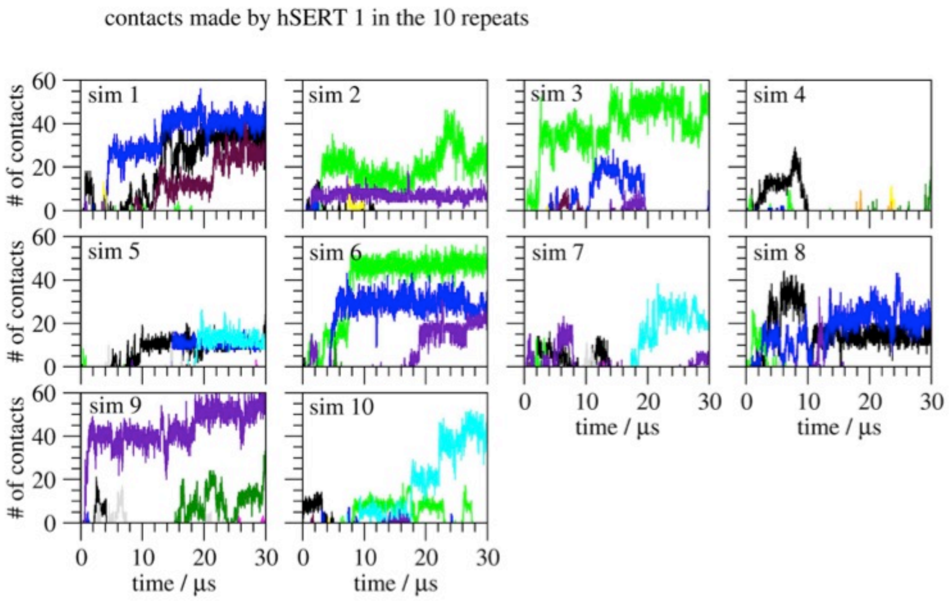

Figure S12. Illustration of the diversity of contacts made by a particular protein in the ten repeats of self-assembly simulations. We show the contacts made by hSERT #1 (top left corner in the 4x4 grid at the start of the simulation) with the other transporters in the repeat (sim) 1 to 10. From ~zero (sim 4) to three (sim 1, 5 and 6) contacts are formed. It is also interesting to see that unbinding events are observed in most cases. Transient interactions are formed for from less than a 1 µs to up to 10 µs (sim 3-blue and sim 4-black).

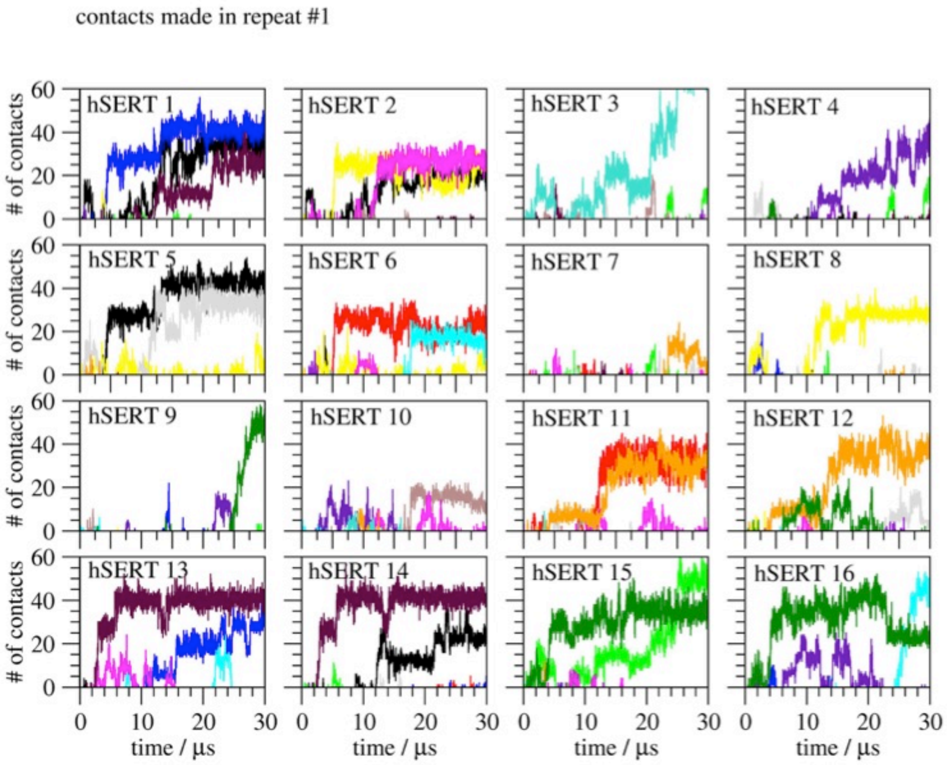

Figure S13. Illustration of the distribution of the numbers of contacts made by a transporter in a particular self-assembly simulation. We show the contacts made in the repeat #1 (top left corner in Figure 1 of the main manuscript) by each of the sixteen transporters. From ~zero (hSERT 7) to three (hSERT 1 and 2) contacts are formed but a majority of transporters form 2 contacts (Figure S1). It is also interesting to see that unbinding events are observed in most cases. Transient interactions are formed for less than a 1 µs to up to 15 µs (contact between hSERT 12 & 16).

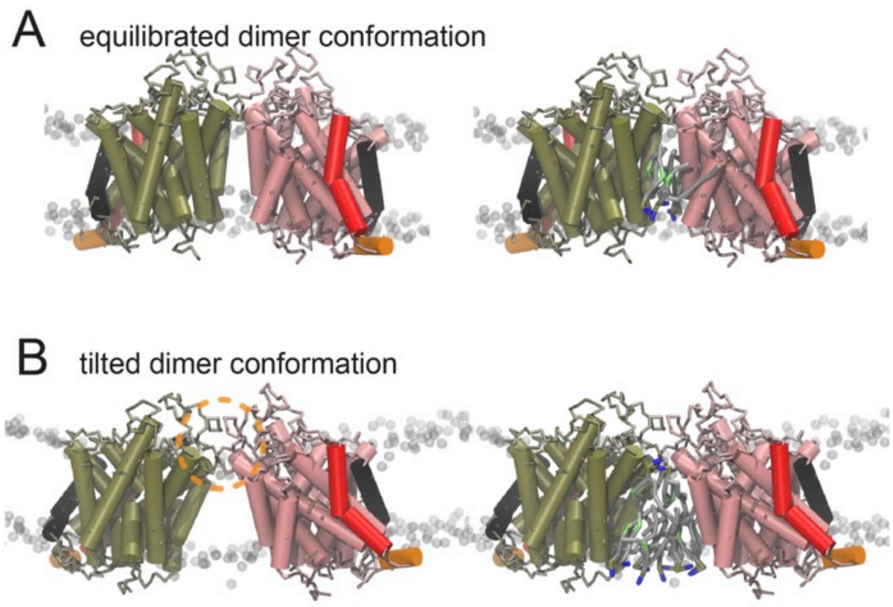

Figure S14. Illustration of dimer configuration of cluster 6. Here we show the conformations A) of the equilibrated bound state (d_{int}=5.8 nm) and B) as found in the umbrella #16 (d_{int}=6.9 nm). The interface is mainly formed in the extracellular part and in the aqueous phase and is highlighted by an orange dashed circle. The lipids at the interface (within 1.4 nm of both proteins) are shown in a stick representation. Lipids are shown only on the right side so the space in between the two proteins might be seen on the left side.

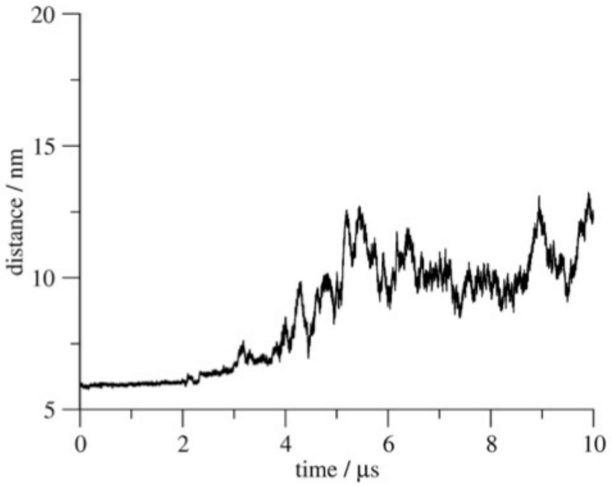

Figure S15. Illustration of the separation of hSERT monomers when allowed to freely evolve (no umbrella potential applied) from a dimer conformation in which they interact. The forces resulting from the interactions between ECL2 of the two monomers are not accounted for. The figure shows the distance between the COMs of the two proteins.

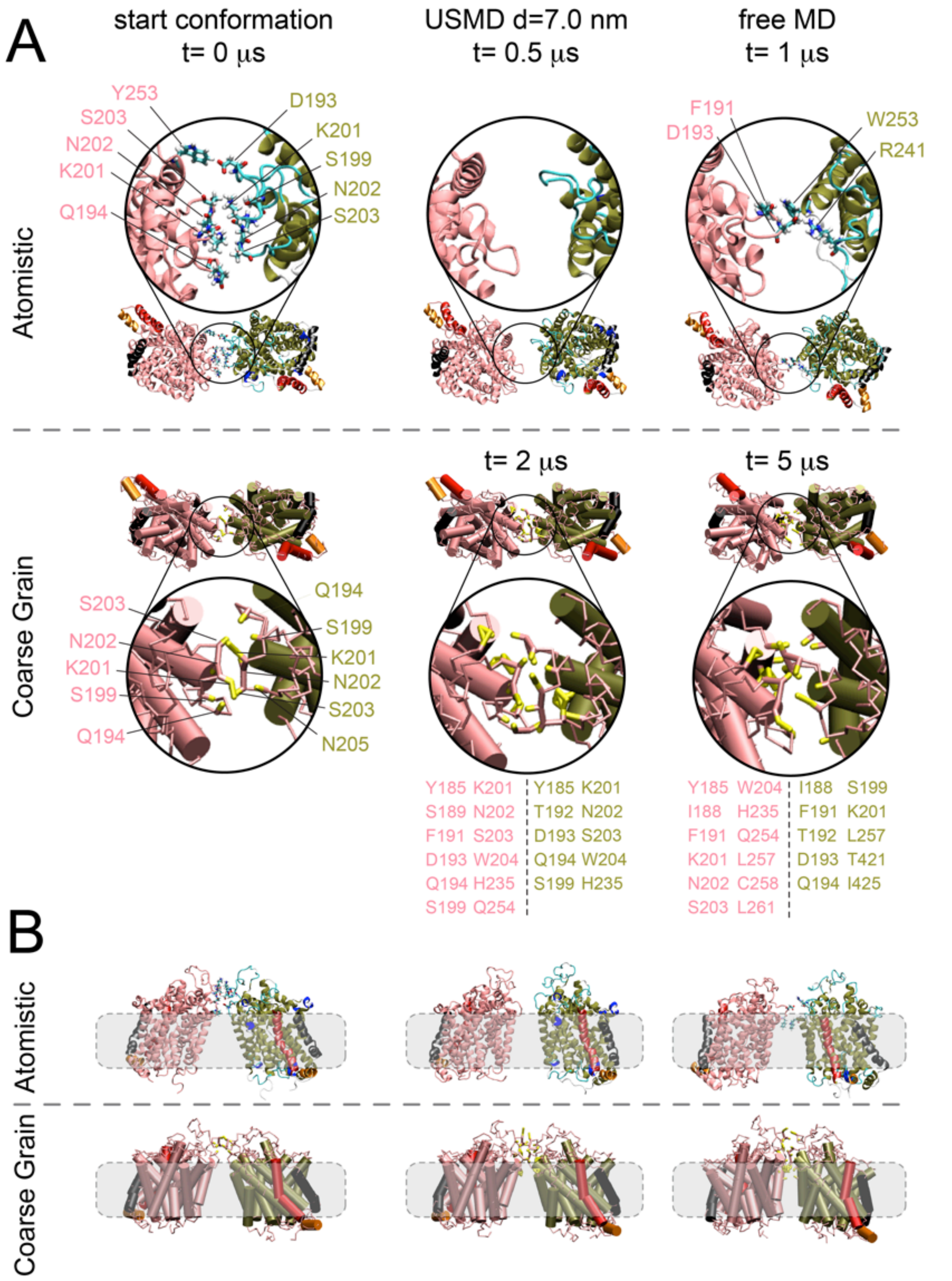

Figure S16. Description of the contacts formed at the interface using the ECL2 at atomistic (AT) and coarse grain (CG) resolutions. The details of the contacts are given in A) with a view from the extracellular side and from the membrane in B). The location of the membrane is represented by a greyed area in B). The interface is described at (left) the start of the simulation (t=0 μs), (middle) after 0.5 and 2 μs, for AT and CG resolutions, respectively, for a simulation using an umbrella potential maintaining the proteins at 7.0 nm, and (right) after 1 and 5 μs, for the AT and CG resolution, respectively, for a simulation where the proteins are left free to evolve. The residues forming a contact are listed for each monomer and each case.

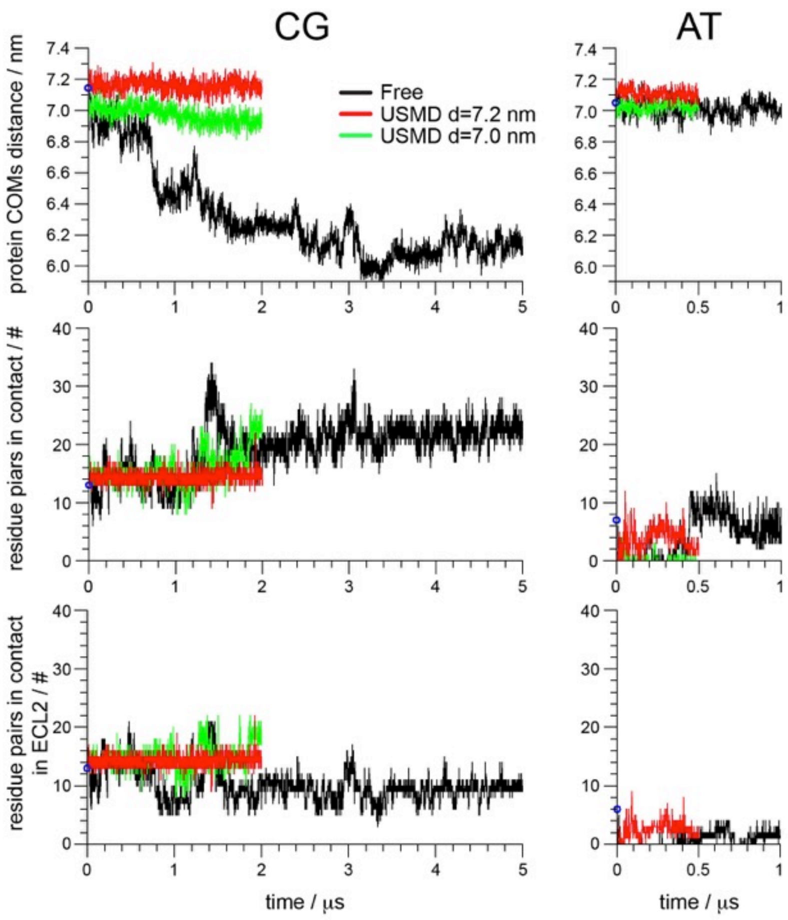

Figure S17. Evolution of the interface involving ECL2 (see Figure S16) in three simulations: unbiased (Free, black), and using an umbrella potential (USMD) to maintain the protein at 7.0 (green) and 7.2 (red) nm. The results of both coarse grain (CG) and atomistic (AT) resolution simulations are presented. The protein center of mass (COM), the number of pairs of residues in contact for all the protein and only counting the ECL2 are shown.

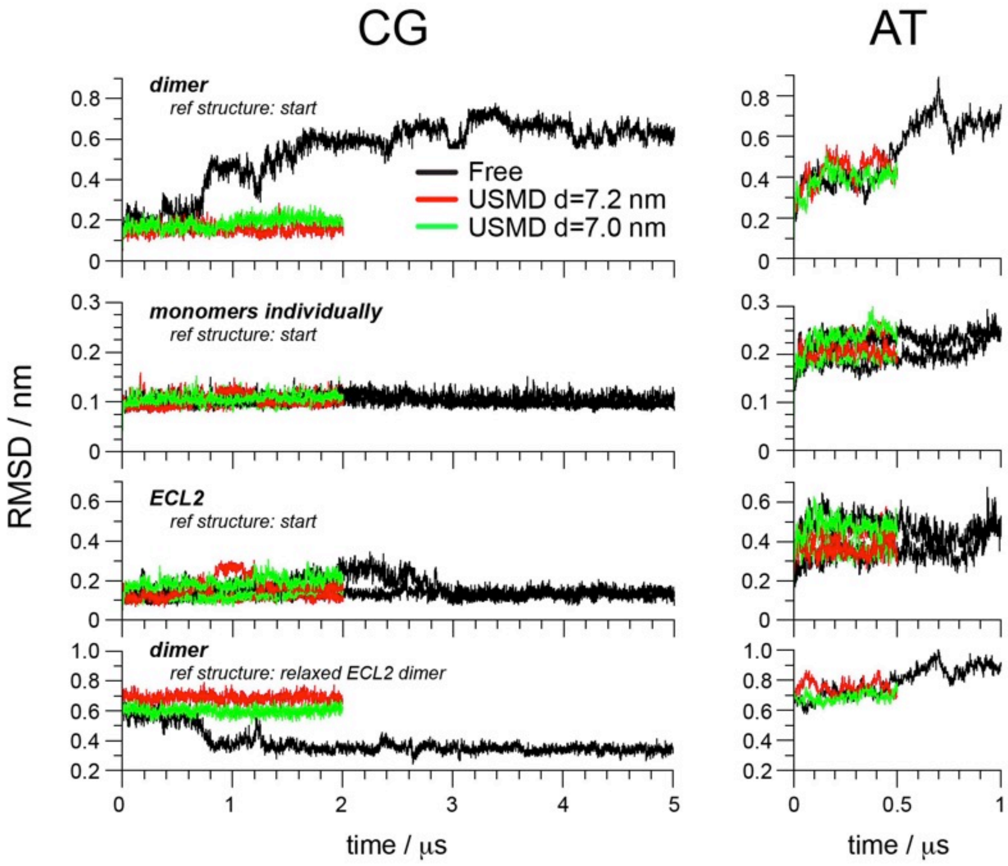

Figure S18. Evolution of the interface involving ECL2 (see Figure S16) in three simulations: unbiased (Free, black), and using an umbrella potential (USMD) to maintain the protein at 7.0 (green) and 7.2 (red) nm. The results of both coarse grain (CG) and atomistic (AT) resolution simulations are presented. The positional root-mean-square deviation of the dimer with reference to the starting structure of the simulation (top), of the monomers after individual fitting to their respective starting structure (second from top), of the extracellular loop 2 (ECL2) after fitting of the entire protein to the starting structure (second from bottom), and of the dimer with reference to the structure of the dimer after equilibration (bottom). The starting structure (tilted proteins) and the equilibrated dimer are shown in Figure S14.

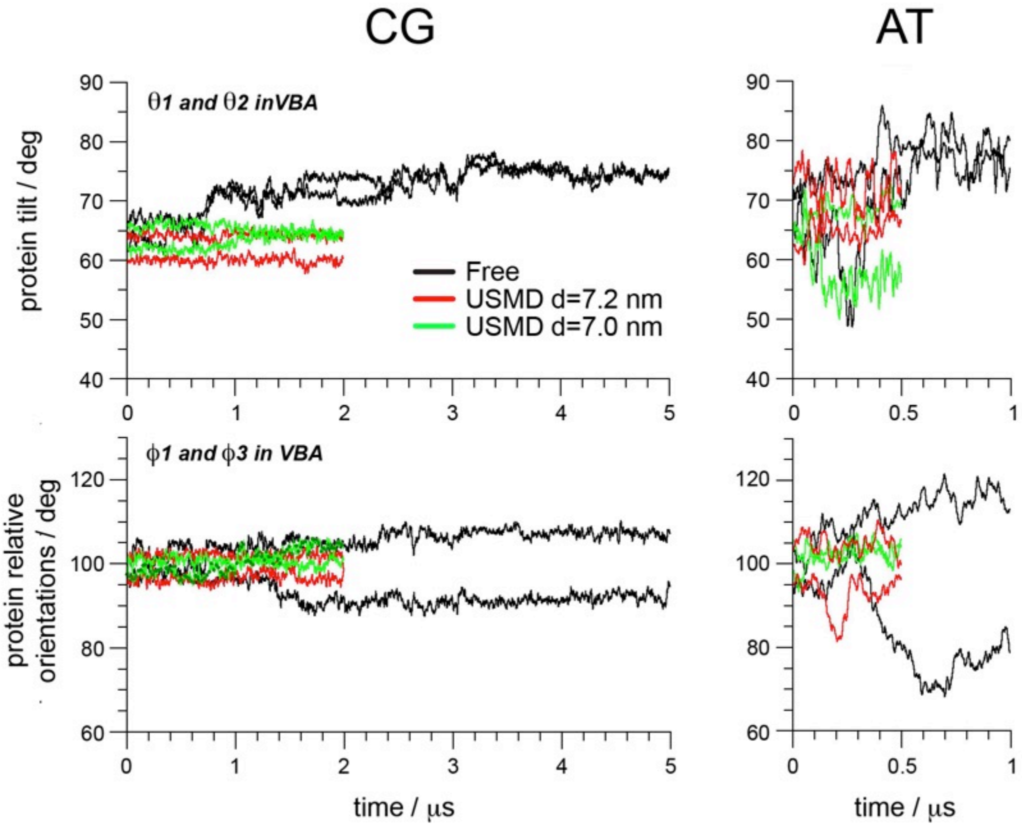

Figure S19. Evolution of the interface involving ECL2 (see Figure S14) in three simulations: unbiased (Free, black), and using an umbrella potential (USMD) to maintain the protein at 7.0 (green) and 7.2 (red) nm. The results of both coarse grain (CG) and atomistic (AT) resolution simulations are presented. The figure shows the tilt of the proteins as depicted by θ1 and θ2 defined by the VBA and the relative orientation of the proteins as depicted by the ϕ1 and ϕ3 dihedral angel of VBA.

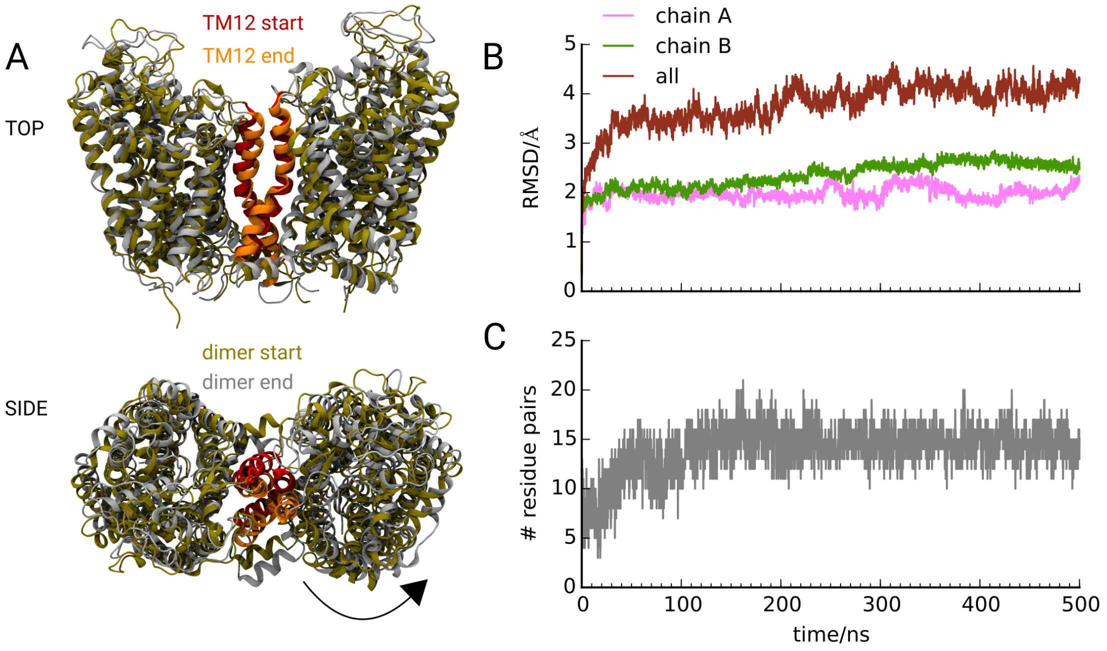

Figure S20. TM12-TM12 dimer stability at atomistic resolution. A) Overlay of the TM12 symmetric dimer before (start) and after (end) a 500 ns simulation. The protein scaffold is colored forest green and grey, respectively, and the TM12 helices are colored red and orange, respectively. The proteins are shown from the top and side with respect to the membrane normal. An arrow indicates a ~10° rotation, which occurs between the two monomers after approximately 50 ns simulation. In B) the root-mean-squared-deviation (RMSD) is presented for each monomer (chain A, pink and B, green) after fitting on the each monomer separately and the whole dimer (all, dark red) after fitting on the whole dimer. In all fitting prior to the RMSD calculation the protein backbone was used excluding the flexible extracellular loop 2 (ECL2, residue 200-230) in chain A and chain B and the whole dimer, respectively. In C) the total number of residue pairs between the two monomers as a function of simulation time is represented. A contact was defined when the minimum distance between two residues was below 5 Å. It is observed that after ~50 ns the number of contacts increases indicating that the rotation of the two monomers with respect to each other (mentioned above) results in an increased number of contacts.

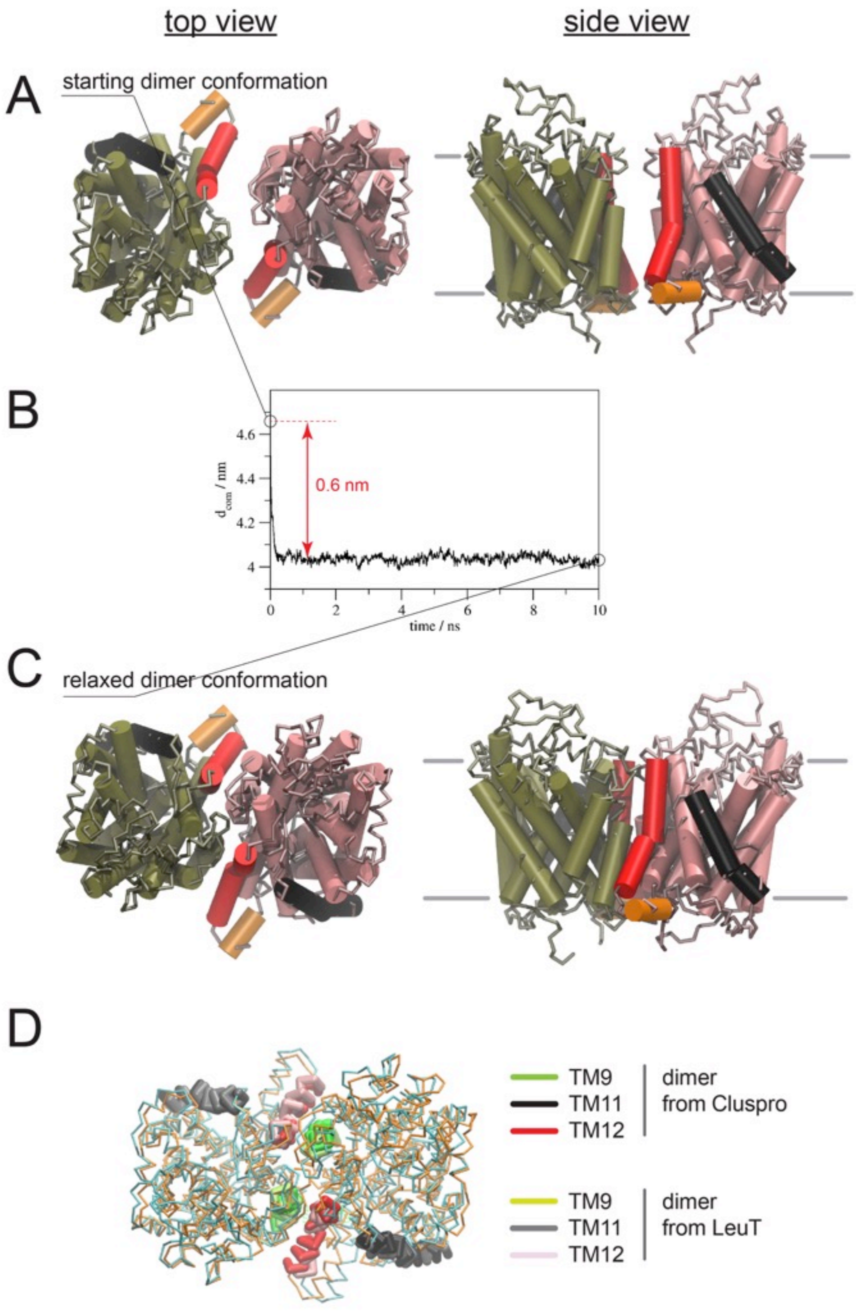

Figure S21. Relaxation of the dimer interface which is based on the conformation found in the LeuT crystal structure . The starting structure in A) relaxes rapidly by reducing the distance by ~0.6 nm B) between the two monomers to form a tight interface C). D) Comparison of the dimer structures obtained using Cluspro and found in the LeuT crystal structure. See main manuscript for more details.

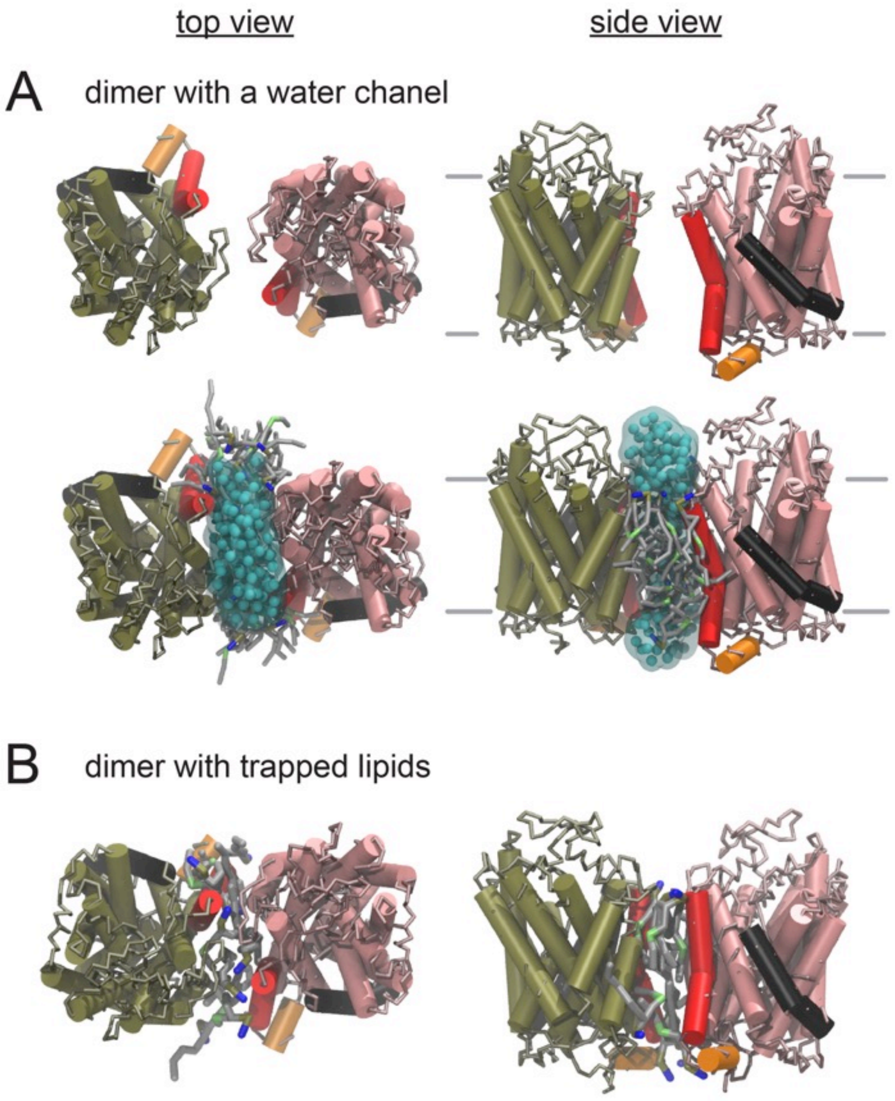

Figure S22. Illustration of the unusual behavior of the interface built following the dimer protein orientation found in the LeuT crystal structure. A) Formation of a water channel at the interface between the proteins as they are separated. The top panel shows the protein without lipids and water molecules, which are illustrated in the bottom panel in gray sticks and blue spheres and transparent surface, respectively. B) Lipids trapped at the interface between the proteins as they are brought together. Lipids are shown in gray sticks. In both A) and B) extracellular (top) and membrane views (side) are given.

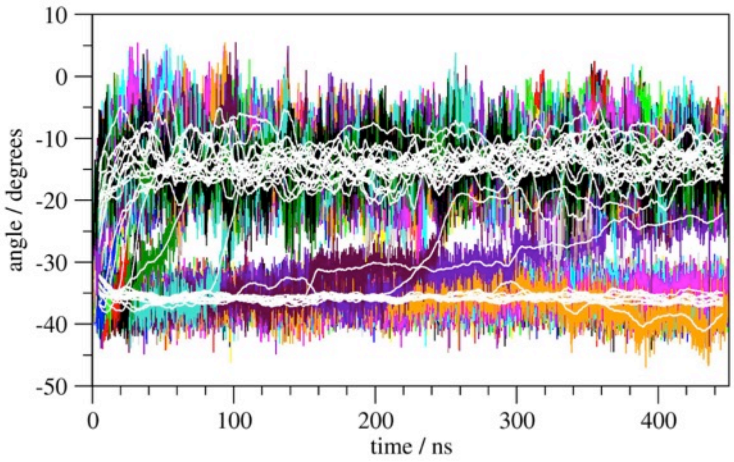

Figure S23. The time evolution of the relative orientation of the two monomers as observed in the US-REMD of the interface of hSERT as found in LeuT crystal structure. The graph shows the BAab angle or ϕ_2 following the VBA notation. The simulation was performed starting with the proteins bound.

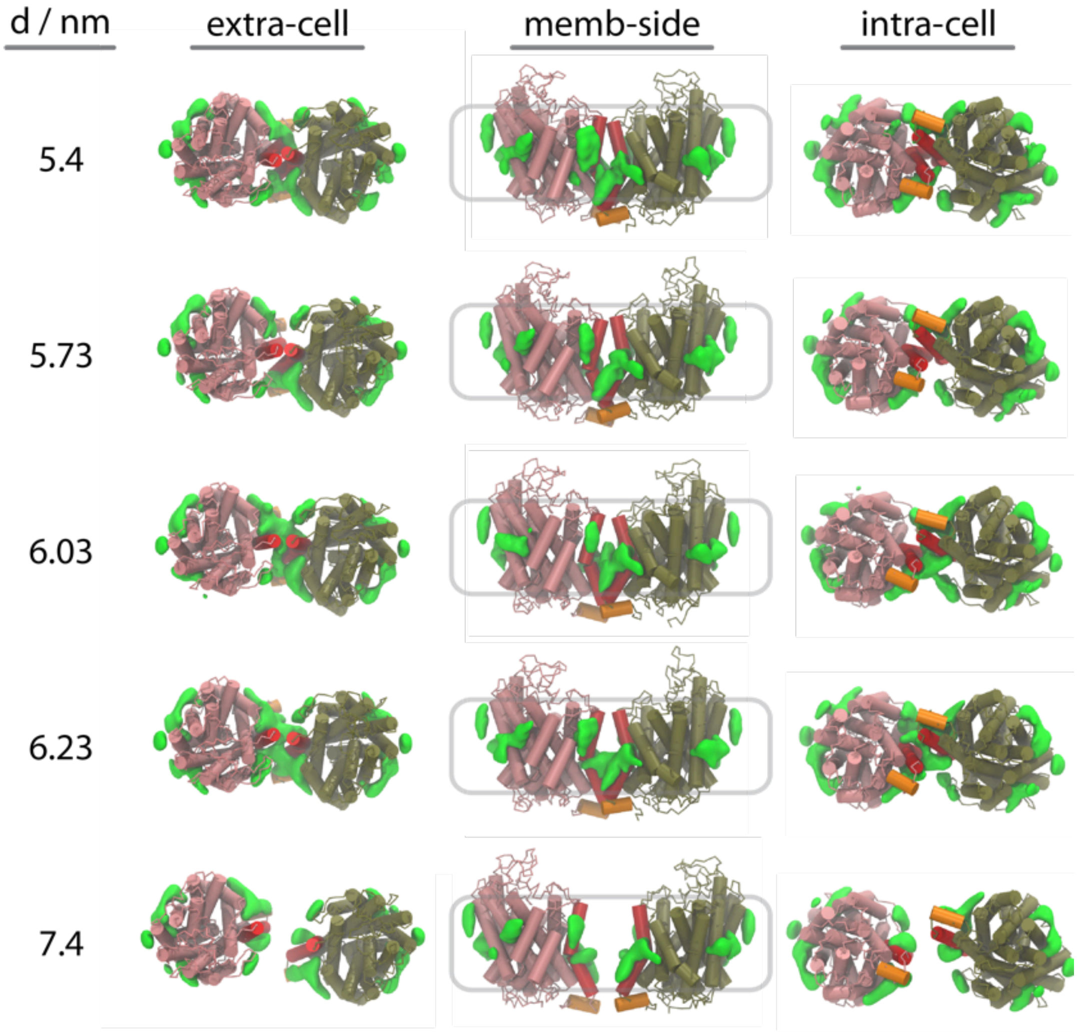

Figure S24. Occupational 3D density maps of cholesterol (green) around hSERT as a function of the distance, d, between the proteins. The distances correspond to the value of the reference for the umbrella potential used in the US-REMD simulation. Therefore it represents the distance between the backbone beads of Leu338 of each monomer. The twelve transmembrane helices are depicted in pink and tan tubes for the two monomers. TM12 and its C terminus are highlighted in red and orange, respectively.

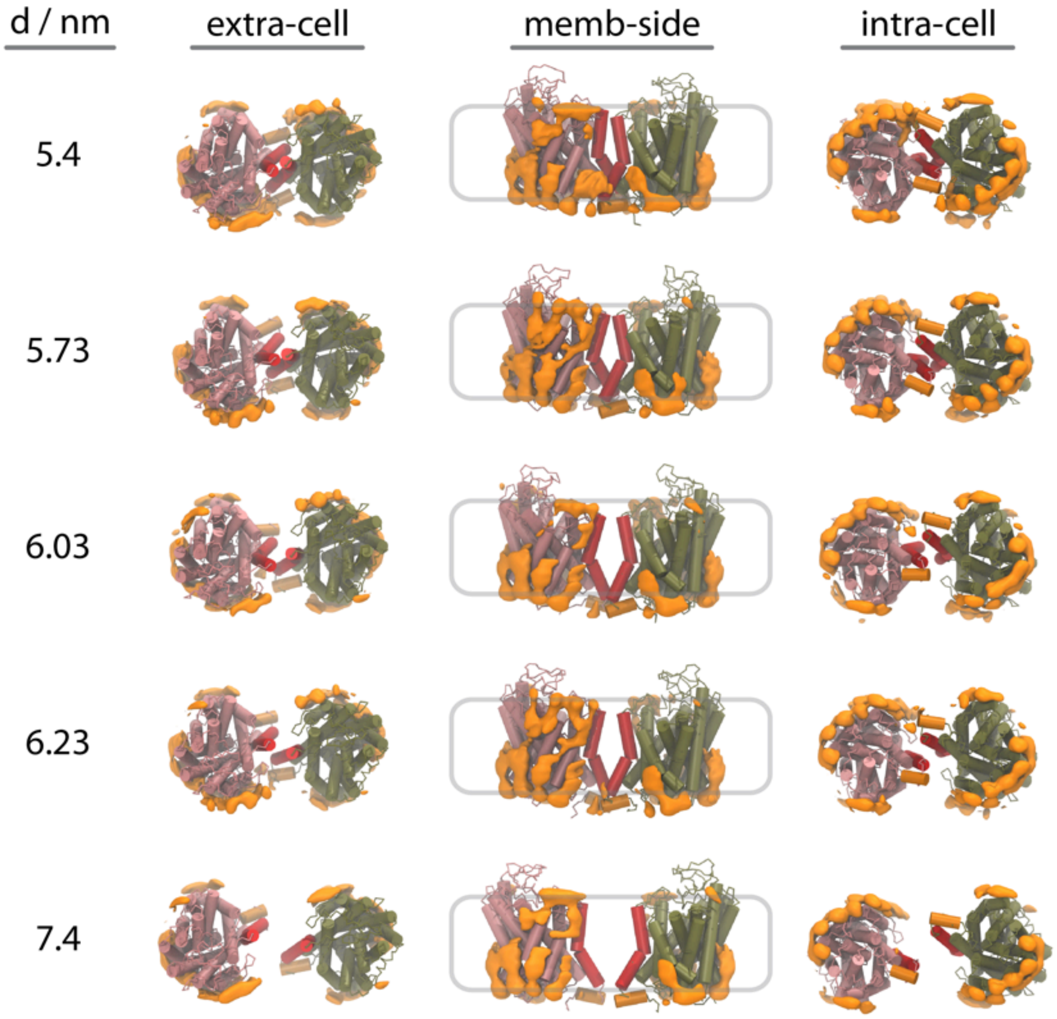

Figure S25. Occupational 3D density maps of POPIP2 (orange) around hSERT as a function of the distance, d, between the proteins. The distances correspond to the value of the reference for the umbrella potential used in the US-REMD simulation. Therefore it represents the distance between the backbone beads of Leu338 of each monomer. The twelve transmembrane helices are depicted in pink and tan tubes for the two monomers. TM12 and its C terminus are highlighted in red and orange, respectively.

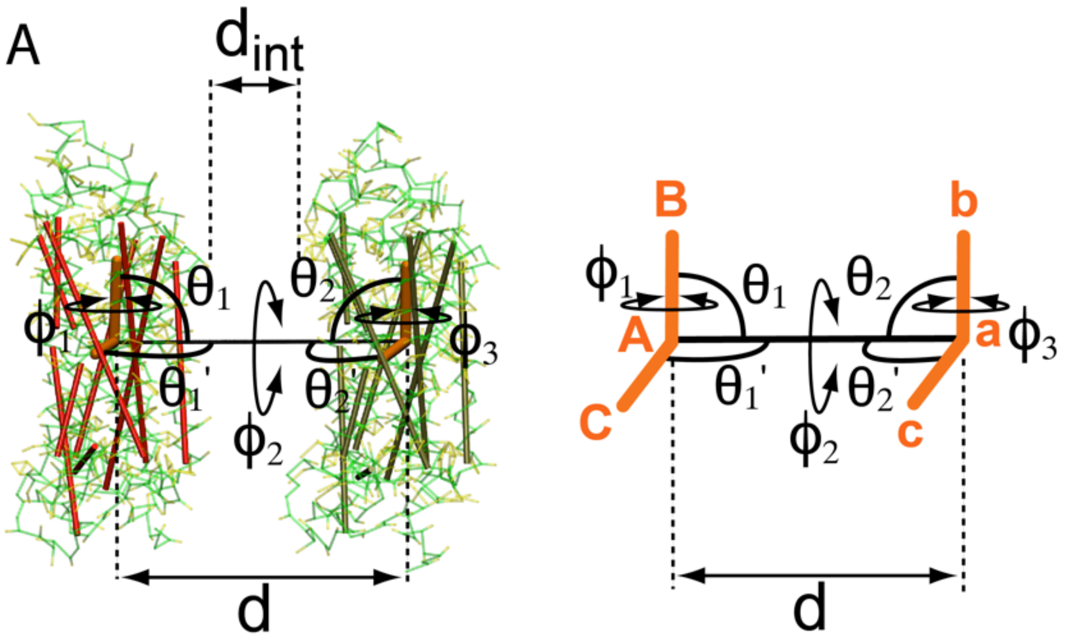

B

Interface	Cluster	Equilibration			VBA	
		d_{eq}	ϕ_1	ϕ_3	ϕ_1	ϕ_3
TM12-TM12	1	5.37±0.05	9.6±2.2	8.6±2.4	9.0	9.0
TM12-TM7	2-3, 9-10	4.54±0.05	5.2±2.0	-165.1±3.1	5.2	-165.1
TM4/9-TM2/11	4-5, 7-8	5.05±0.05	85.5±2.1	-102.7±3.6	85.5	-102.7
TM3/4-TM3/4	6	5.83±0.05	100.6±2.2	102.9±2.2	101.8	101.8
hSERT on LeuT	-	5.19±0.05	37.0±2.2	42.6±1.8	40.0	40.0

Figure S26. Virtual bond algorithm. **A**) Schematic representation of the virtual bond algorithm (VBA) used to define and control the relative orientation of the protein while performing a potential of mean force. In the current case (hSERT) the anchors A/a, B/b and C/c were chosen as the backbone bead of Leu338, Gly402 and Val501, respectively. **B**) Parameters used in the VBA (panel **A**) to describe the different interfaces. For each interface the corresponding info is given: the cluster(s) to which it belongs, averaged values extracted from an MD simulation of a representative structure of the cluster for the distance d, and the angles ϕ_1 and ϕ_3 used to define the relative orientation of the proteins. See panel **A** for details of the parameters.
